# Context-Sensitive Spelling Correction of Consumer-Generated Content on Health Care

**DOI:** 10.2196/medinform.4211

**Published:** 2015-07-31

**Authors:** Xiaofang Zhou, An Zheng, Jiaheng Yin, Rudan Chen, Xianyang Zhao, Wei Xu, Wenqing Cheng, Tian Xia, Simon Lin

**Affiliations:** ^1^ The Department of Ophthalmology Wuhan Central Hospital Wuhan China; ^2^ Internet Technology and Engineering Research and Development Center School of Electronic Information and Communications Huazhong University of Science and Technology Wuhan China; ^3^ Department of Biostatistics and Computational Biology School of Life Science Fudan University Shanghai China; ^4^ Northwestern University Biomedical Informatics Center (NUBIC) Feinberg School of Medicine Northwestern University Chicago, IL United States; ^5^ Research Institute Nationwide Children's Hospital Columbus, OH United States

**Keywords:** spelling correction system, context sensitive, consumer-generated content, biomedical ontology

## Abstract

**Background:**

Consumer-generated content, such as postings on social media websites, can serve as an ideal source of information for studying health care from a consumer’s perspective. However, consumer-generated content on health care topics often contains spelling errors, which, if not corrected, will be obstacles for downstream computer-based text analysis.

**Objective:**

In this study, we proposed a framework with a spelling correction system designed for consumer-generated content and a novel ontology-based evaluation system which was used to efficiently assess the correction quality. Additionally, we emphasized the importance of context sensitivity in the correction process, and demonstrated why correction methods designed for electronic medical records (EMRs) failed to perform well with consumer-generated content.

**Methods:**

First, we developed our spelling correction system based on Google Spell Checker. The system processed postings acquired from MedHelp, a biomedical bulletin board system (BBS), and saved misspelled words (eg, sertaline) and corresponding corrected words (eg, sertraline) into two separate sets. Second, to reduce the number of words needing manual examination in the evaluation process, we respectively matched the words in the two sets with terms in two biomedical ontologies: RxNorm and Systematized Nomenclature of Medicine -- Clinical Terms (SNOMED CT). The ratio of words which could be matched and appropriately corrected was used to evaluate the correction system’s overall performance. Third, we categorized the misspelled words according to the types of spelling errors. Finally, we calculated the ratio of abbreviations in the postings, which remarkably differed between EMRs and consumer-generated content and could largely influence the overall performance of spelling checkers.

**Results:**

An uncorrected word and the corresponding corrected word was called a spelling pair, and the two words in the spelling pair were its members. In our study, there were 271 spelling pairs detected, among which 58 (21.4%) pairs had one or two members matched in the selected ontologies. The ratio of appropriate correction in the 271 overall spelling errors was 85.2% (231/271). The ratio of that in the 58 spelling pairs was 86% (50/58), close to the overall ratio. We also found that linguistic errors took up 31.4% (85/271) of all errors detected, and only 0.98% (210/21,358) of words in the postings were abbreviations, which was much lower than the ratio in the EMRs (33.6%).

**Conclusions:**

We conclude that our system can accurately correct spelling errors in consumer-generated content. Context sensitivity is indispensable in the correction process. Additionally, it can be confirmed that consumer-generated content differs from EMRs in that consumers seldom use abbreviations. Also, the evaluation method, taking advantage of biomedical ontology, can effectively estimate the accuracy of the correction system and reduce manual examination time.

## Introduction

### Background

In the last two decades, spelling correction methods for clinical texts have been studied extensively. Nevertheless, the majority of related studies mainly focused on the electronic medical record (EMR) [1], but largely ignored consumer-generated content which has accumulated rapidly because of the development of online media and social networks. The consumers mentioned here include those who describe their symptoms and seek online medical assistance, and those who have been successfully cured and willing to share their treatment process experience on public websites or forums. Although there is no doubt that the EMR content is worthy of in-depth study, information in consumer-generated content is equally useful and informative, which has been discussed in a US National Research Council Committee Framework [2] and in Zeng et al [3]. Mining information in consumer-generated content based on large-scale text analysis becomes increasingly important in the context where social networks have become pervasive in recent years. For example, the useful relationship information between biomedical terms can be inferred based on texts extracted from postings in various online health communities written by patients. Obviously, the accuracy of these inferences relies on correctly spelled text. Therefore, the development of spelling correction methods for consumer-generated content is critical for ensuring the accuracy and efficiency of downstream text analysis.

### Related Work

#### Spelling Correction

Numerous approaches for correcting spelling errors, such as Levenshtein edit distance [4,5] and semantic correction [1,6], have been proposed. The Levenshtein edit distance model demonstrates a method to measure the edit distance of converting one string to another, which is calculated by counting the number of four-letter operations—deletions, insertions, transpositions, and substitutions—during the conversion. For example, when correcting “plls” to “pills,” we need to insert the letter “i” which increases the edit distance by one. The candidate with the lowest edit distance will be recognized as the best replacement for the misspelled word. The semantic correction model utilizes context-sensitive detection and has been widely applied to studies using natural language processing (NLP). For example, Wong and Glance [1] developed a robust system using semantic correction to correct misspelled words, especially abbreviation disambiguation, in progress notes. In addition, according to a study proposed by Ruch et al [7], these two models can be combined: first, the Levenshtein edit distance is computed and the resulting candidate words are ranked according to the edit distance. Each word is then examined according to the context using semantic correction. Finally, the best suitable candidate is picked according to both edit distance and semantic meaning. There are other extensively used methods such as the Soundex system proposed by Odell and Russell [8,9] and the n-gram model [7,10,11]. Some studies applied an integrated spelling correction application programming interface (API), such as GNU Aspell, Yahoo API, etc. Wong and Glance [1] adopted and mixed GNU Aspell and Yahoo API corrective interfaces in their systems for real-time abbreviation disambiguation, which has achieved good results. These interfaces have become highly sound and mature after a long period of development.

#### Evaluation Methods

The mainstream evaluation methods for spelling correction systems can be ascribed into two types: horizontal comparison and longitudinal comparison. Horizontal comparison means that researchers test several different correction models with the same input, and then compare their performance and accuracy to prove the strength of the newly designed model. For example, in the study by Ruch et al , they compared the correction results of four different correction models derived from NLP. Longitudinal comparison is generally applied in evaluating methods which are used to improve and perfect existing spelling correction systems. This comparison mainly focuses on the difference between the spelling error correction rate before and after the improvement, as in Crowell et al [12].

Currently, most of the prevalent evaluation methods for spelling correction are based on manual inspection [1]. Although it is accurate, the manual evaluation is time consuming, and not feasible to be applied in large-scale experiments. Therefore, we explored the use of formal ontologies to evaluate the effectiveness of spelling correction.

#### Spelling Error Classifications

Spelling errors are usually divided into different categories. Ruch et al classified misspelled words in EMRs into two categories. The first category, called typographical error, refers to spelling mistakes which lead to misspelled words becoming nonexistent in the dictionary. For instance, when a consumer spells “plls” instead of “pills,” there is no chance of finding “plls” in a lexicon. The second category, called *linguistic error*, refers to typing errors which cause a word’s original meaning to change, but the misspelled word still exists in the dictionary (eg, spelling “three pills” as “tree pills”). Syntactic and semantic spelling errors are included in this category. Similarly, many other studies, such as those of Jurafsky and James [13] and Wilbur et al [14], classified spelling errors according to whether misspelled words needed *isolated-word error correction* or *context-dependent error correction*. Our system followed Ruch’s classification method, categorizing spelling errors into *typographical* and *linguistic errors*.

### Limitations With Existing Approach

There are several limitations that exist within the current approaches and hinder the correction process from achieving highly efficient performance. To begin with, some existing approaches will become less efficient and require an abundance of training data when processing large amounts of text. For example, according to Ruch et al, correction systems using Levenshtein edit distance require extremely large amounts of training data, which can be scarcely satisfied in real-world situations. Also, the semantic correction process is highly complex when the correction system needs to detect both typographical and linguistic errors [7].

In addition, the context-related errors make up a large ratio of spelling errors in consumer-generated content (shown in the following sections). If we only focus on typographical errors [15] in order to achieve high efficiency, then the accuracy of the correction system will be largely sacrificed, and overall system performance will appear much less desirable than approaches considering both linguistic and typographical errors.

Moreover, unique features of consumer-generated content should also be taken into consideration in the correction process. Consumer-generated content differs from EMR content, in that there are many abbreviations written by clinical professionals in EMRs, which are rarely shown in consumer-generated content. EMRs contain abbreviated terms such as “VSS” (vital signs stable), “PVCs” (premature ventricular contractions), ‟NTG” (nitroglycerin), and ‟gtt” (guttae) to describe patients’ physical and mental conditions in a quantitative and professional fashion, while consumers prefer to describe their conditions using common language such as “depressed,” “pain,” and “feel better.” This distinct feature leads to differences in spelling correction strategies between EMR and consumer-generated content [1].

### Our Approach

We proposed a spelling correction system based on Google Spell Checker, which is not only able to automatically correct both typographical and linguistic errors, but is also highly efficient thanks to Google Spell Checker’s core algorithms [16]. Our system focuses on correcting spelling errors in daily medical vocabularies, rather than professional, but not commonly used, terminology like the methods proposed by Wang et al [1], Doan et al [17], and Patrick et al [18]. It is a real-time and high-performance method that can be easily applied to studies requiring automatic correction of misspelled words.

In order to shorten the evaluation period and preserve the reliability of the evaluation, we narrowed down the range of words being examined by matching these words with biomedical ontology items, and then manually examining the matched words. Ontologies consist of words and phrases describing and annotating concepts in many fields, such as biomedical informatics * and artificial intelligence. To evaluate our system, we selected two* biomedical ontologies: Systematized Nomenclature of Medicine -- Clinical Terms (SNOMED CT), which is focused on diseases and symptoms, and RxNorm, which is focused on drugs.

## Methods

### Dataset

**Figure 1:**
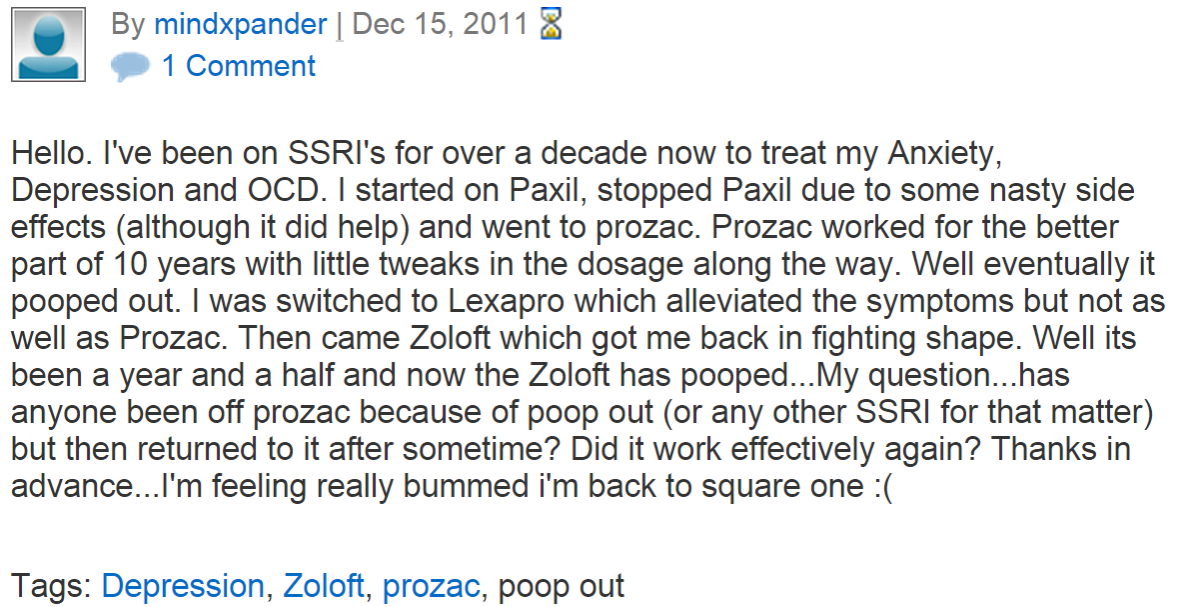
Screenshot of a sample post from MedHelp's bulletin board system.

In this study, we randomly selected 150 postings (21,358 words in total; Multimedia Appendix 1) from MedHelp’s bulletin board system (BBS) [19]. This set of postings is related to a drug named *Zoloft* and contains consumers’ descriptions of their symptoms and suggestions from others, such as doctors, pharmacists, and patients, who have already used Zoloft. Figure 1 shows one example from the 150 postings.

### Tools Used in Our Study

#### Google Spell Checker

We based our system on Google Spell Checker, a state-of-art spelling correction tool which is embedded in Google Search and utilizes the Web pages as corpus. Our system can upload text segments, which need spelling checked, onto Google Search and spelling suggestions will be automatically generated by Google Spell Checker. Google Spell Checker’s high accuracy and efficiency have been proven by Jacquemont et al and Islam and Inkpen who applied Google’s search engine and Google Web 1T n-gram—a language model extracting nearly 1 trillion words from Web pages—into the spelling correction process.

#### National Center for Biomedical Ontology Annotator

To reduce the amount of manual work in the evaluation process, we used the National Center for Biomedical Ontology (NCBO) Annotator [20] to match texts with formal ontologies. The NCBO is a website which contains all biomedical ontologies and relevant knowledge; ontology is a set of terms related to a certain subject, such as biochemistry and movement (eg, “*Amino Acid Ontology*” and “*Cell Ontology*”). The NCBO’s Annotator is used to search annotations of biomedicine-related texts in the given ontologies. After selecting ontologies and submitting original texts, users will obtain matched terms from the Annotator; terms exist in the designated ontologies. In addition, there is no need for the users to manually submit text one by one in NCBO’s website. A Web service is provided for all users to accomplish the text-mining jobs programmatically [21].

### Framework

#### Construction of Our Spelling Correction System

We developed our spelling correction system based on Google Spell Checker. The system works in three steps: text segmentation, text spelling correction, and text reconstruction.

In the first step—text segmentation—content (eg, a post from MedHelp) is automatically grouped into sets of less than 32 words, since Google’s search engine can only process 32 words at a time in the correction program. It is worth mentioning that, although our system divides the postings automatically, it does not destroy the complete structure of one sentence. According to the online data [22], the average sentence length is 15 to 20 words, which is less than the 32-word requirement in the Google search engine. Additionally, the Google Spell Checker is able to consider the context of the candidates’ suggestions, and evolves in accordance with the update of millions of Web pages [16]. All the segments processed are saved in our database. In this way, when context-sensitive texts are separated, this will prevent changes to their original meaning.

In the second step—text spelling correction—our system uploads the segments saved in the database onto Google Search and downloads the feedback generated by Google Spell Checker. Google Spell Checker not only corrects typographical errors but also proposes suggestions for linguistic errors according to relations of context, including syntactic and semantic relations. The syntactic relation helps in correcting grammatical errors. For example, in some posts, “*had*” was misspelt as “*has,*” but it turns out that “*had*” was more suitable in the contexts. In these circumstances, our system can find this type of problem and deliver the correct output. The semantic relation is used in correcting consumer mistakes that may produce ambiguity (eg, mistakenly writing “*three*” as “*tree*”). These problems can be resolved using the Google Spell Checker because it can intelligently conclude the most probable text candidate according to the sentence meaning. After correcting the whole text, the system will output and save the corrected text. Table 1 uses the sentence “I tooj tree pills last night before bad time” as an example, and explains how our system works on sentences. Each row shows how our system corrects a single word each time. The number in the second row, such as “-1” and “+3,” shows the position of each word in this sentence. For example, if we are presently focusing on the word “tooj,” then “-1” corresponds to “I” and “+3” corresponds to “last.” The column “Correction” shows the corrected results. The last column, “Error type,” is manually classified, which will be discussed in the Error Classification section.

In the third step—text reconstruction—our system reconstructs full-text segments in accordance to their original order. Throughout the above three-step operation, our system will successfully correct the input postings and save both the uncorrected and corrected texts into our database.

Thus, we entered the consumer-generated postings collected from MedHelp and followed the steps above. After the correction process, we obtained both misspelled and corresponding corrected words, *respectively* saved into the uncorrected (U) *set and the corrected (C) set*. For example, after processing the sentence “I tooj tree pills last night before bad time,” “tooj,” “tree,” and “bad” will be saved in set U and “took,” “three,” and “bed” in set C.

**Table 1:** The spelling correction process.

Step number	Word position^a^			Misspelled word	Word position^a^			Correction	Error type
	-3	-2	-1		+1	+2	+3		
1			I	tooj	tree	pills	last	took	typographical
2		I	tooj	tree	pills	last	night	three	linguistic
3	last	night	before	bad	time			bed	linguistic

^a^The number represents the position of each word in the sentence relative to the word presently being focused on.

#### Evaluation Process

**Figure 2:**
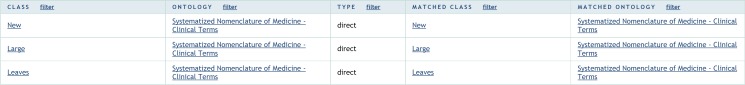
Screenshot of the NCBO Annotator presenting words before spelling correction.

**Figure 3:**
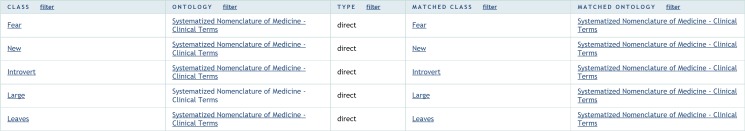
Screenshot of the NCBO Annotator presenting words after spelling correction.

During the evaluation of our system’s correction quality, first we used the NCBO Annotator Web service to decrease the number of words examined manually; we input corrected words from set C into the NCBO Annotator, selected the RxNorm and SNOMED CT ontologies, and then ran the Annotator search. The reason we selected these two ontologies was because the former, RxNorm, contains all of the terminologies of drugs available on the US market [23], and the latter, SNOMED CT, contains a collection of clinical terms and is recognized as the most comprehensive health care terminology resource in the world [24]. After the data had been completely scanned and processed, the NCBO Annotator presented the words which could be matched in RxNorm and SNOMED CT in a downloadable Web page (see Figure 2). We then downloaded and saved the matched words. Similarly, we also input uncorrected words from set U, acquired the words which could be matched in RxNorm and SNOMED CT, and then saved them into our database (see Figure 3). After this preprocessing, instead of examining all the words in set C and set U, we could only manually examine the matched words, count the number of words which were appropriately corrected, and then calculate the ratio of these corrections.

#### Error Classification

We manually classified the results into two sets of errors—typographical errors (set T) and linguistic errors (set L)—and invited two clinical doctors and a medical researcher to confirm the correctness of our classification.

#### Abbreviation Counts

In accordance with the definition in Wong and Glance [1], abbreviations in this study refer to shortened forms of words, including acronyms, initialisms, and so on. Following this definition, we manually counted the number of abbreviations in the postings.

## Results

Our spelling correction system detected 271 spelling errors in the selected postings (see Multimedia Appendix 2). For ease of explanation, we called an uncorrected word and its corresponding corrected word a spelling pair, and the two words in the spelling pair are its members. For example, “tooj” and “took” compose, and are the members of, the spelling pair. A total of 271 spelling pairs were detected, among which we found that 58 (21.4%) spelling pairs contained one or two matched members in the selected ontologies—a member able to be matched in the ontologies is called a matched word, and its pair is called a matched pair (see Multimedia Appendix 2). We ascribed the 58 matched pairs into two groups—positive and negative impact—to evaluate the accuracy of our system. Positive refers to misspelled words corrected appropriately and negative refers to those corrected inappropriately. The two impacts contain several different situations and their definitions are shown in Table 2.

In the correction process, we respectively recorded the number of situations defined above through manual inspection (see Table 3) and we asked three senior medical professionals—two clinical doctors and a medical researcher—to verify the correctness our classification.

**Table 2:** Definition for positive and negative impacts.

Impact	Situation	Definition
**Positive**		
	New match identified	Words cannot be found in the ontology before correction, but can be found after correction, and the corrected word is suitable in context.
	Wrong match identified	Words can be found in the ontology before correction, and cannot be found after correction, but the uncorrected word is unsuitable in context.
	Better match identified	Both words before and after correction can be found in the ontology and the corrected word is more suitable in context.
**Negative**	Right match missed	Either or both words before and after correction can be found in the ontology, but the corrected word is inappropriate in context.

**Table 3:** Results of spelling correction experiment (n=58).

Impact	Example	Effect	Ontology, n (%)	Representative letter
New match identified/ positive	“converts to seretonin” → “converts to serotonin”	A match of “serotonin” is found	37 (64)	A
Wrong match identified/ positive	“I took tree pills” → “I took three pills”	The improper match of “tree” is avoided	8 (14)	B
Better match identified/ positive	“last night before bad time” → “last night before bedtime”	A better match of “bedtime” replaces “bad time”	5 (9)	C
Right match missed/ negative	“I'm no chemist” → “I'm no chemistry”	A wanted match of “chemist” disappears	8 (14)	D
Total			58 (100)	F

The first column gives the situations that we defined in Table 2, including detailed types and their impact. The second column shows one example for each situation about how our system corrects the spelling errors. The third column explains the effect of the correction process on the sentences. The fourth column presents the number of matched pairs that conform to the corresponding situation. The letters in the fifth column represent the corresponding number in the fourth column (ie, A=37, B=8, C=5, D=8, and F=58).

From the results, it shows that 64% (A divided by F, 37/58) of the words could not be found in the ontology before correction, could be found in the ontology after correction, and the corrected words were suitable in the context by the situation definition.

Similar to Wong and Glance [1], we calculated the following expression to explain the performance of this system:

Accuracy = (A+B+C)/F

**Figure 4:**
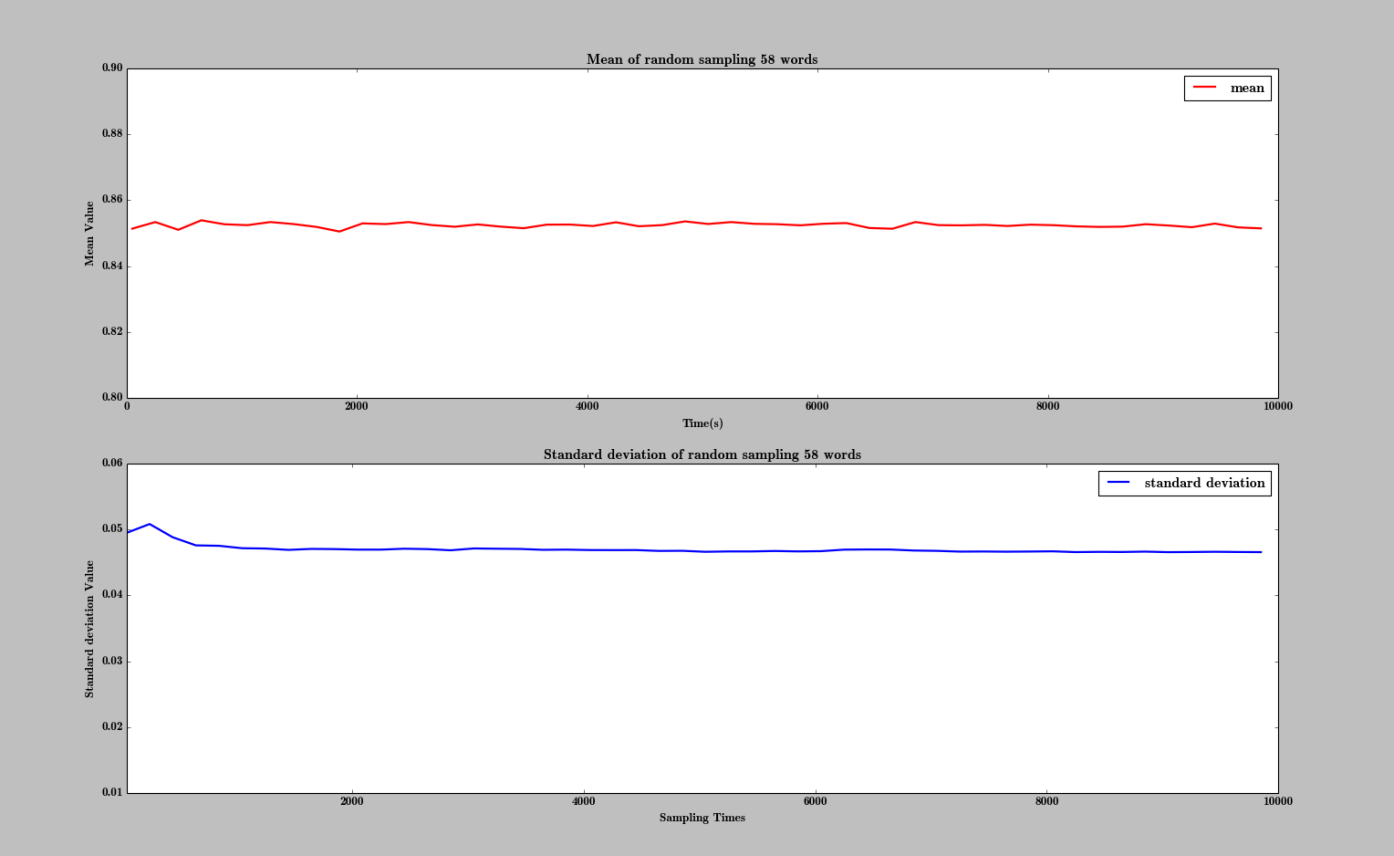
Trends of mean value and standard deviation with change in sample size.

The accuracy—the ratio of misspelled words appropriately corrected in the 58 spelling pairs—was 86% (50/58). Also, we calculated the ratio of appropriate correction in the 271 overall spelling errors to be 85.2% (231/271). We also did a series of random sampling experiments; we randomly sampled 58 spelling pairs each time from the 271 spelling pairs. The trends of mean value and standard deviation are shown in Figure 4. The figure shows that, as the number of experiments increased, both the trends of mean value and standard deviation gradually became stable, respectively approaching 85.3% and 0.047.

After the classification according to the types of spelling errors, from a total of 271 errors our system detected 186 (68.6%) typographical errors (saved in set T) and 85 (31.4%) linguistic errors (saved in set L). In addition, there were a total of 210 abbreviations, making up 0.98% of all words in the postings (n=21,358).

## Discussion

### System Performance

From the correction results, we found that 64% (37/58) of the matched words were newly found, which proved that our correction process exerted a positive effect on increasing the accuracy of downstream biomedical research, such as NLP research. Using the same corpus, our system’s accuracy (50/58, 86%) was higher than that of most of the commonly used spelling checkers, including medical dictionary-based Aspell [25], Microsoft Office Word 2013, and Jazzy Spell Checker [26]. The result is shown in Table 4 and the detailed data are included in Multimedia Appendix 2. This illustrates that our spelling correction system is a suitable and high-performance tool for consumer-generated content.

**Table 4:** Comparison of spell checking tools for finding correct words for misspelled words.

Spell checking tool	Correct words found, n/n (%)
Our method	50/58 (86)
Aspell [25] with general dictionary	304/763 (39.8)
Aspell [25] with medical dictionary	353/564 (62.6)
Microsoft Office Word 2013	313/431 (72.6)
Jazzy Spell Checker [26]	240/574 (41.8)

It is noteworthy that, compared with other spelling checkers which usually provide several spelling suggestions to choose from for a spelling error, our method is more convenient and can directly provide the optimal candidate according to its context. In addition, in contrast to traditional spelling checkers such as Aspell, the corpus in our method does not need manual updates due to its Web page-based corpus. These characteristics are highly meaningful, especially for the automatic spell checking of big data.

Moreover, unlike the method applied by Ruch et al [7] in which spelling errors were artificially added into spelling error-free texts, our system obtained the original text directly from a health forum, which more objectively reflected the real situation of consumer-generated content.

### Classification

From the classification results of spelling error types, it can be observed that errors in set L took up 31.4% of all spelling errors, which shows that correcting linguistic errors is indispensable during processing consumer-generated content. The systems that only focused on the correction of typographical errors, such as that of Peterson [15], ignore a large number of the spelling errors.

### The Number of Abbreviations

In EMRs, the ratio of abbreviations is 33.6% [1], much higher than the ratio in consumer-generated content (0.98%). Therefore, detecting and correcting abbreviations in consumer-generated content appears to be much less important than in EMRs. Instead, from the results of classifying 271 spelling errors according to the meaning of corrected words (see Table 5), the correction systems for consumer-generated content should focus more on common vocabularies.

In Table 5, common vocabulary refers to those words people frequently use in daily life (eg, “good,” “hadn’t,” and “loose”). Medical vocabulary refers to words that cannot be defined as a symptom, drug, or disease but are still used in the medical field such as “hygiene.” Extra space refers to situations in which consumers enter extra spaces between words (eg, “weight__is” where there are two spaces between “weight” and “is”).

**Table 5:** Classification of misspelled words (n=271).

Type of word, or issue	Number of words, n (%)
Common vocabulary	151 (55.7)
Symptom	8 (3.0)
Drug	12 (4.4)
Medical vocabulary	14 (5.2)
Disease	1 (0.4)
Extra space	85 (31.4)

### Evaluation

During the evaluation process, only 58 words matched in the ontology, which was only about one-fifth (21.4%) of the number originally needed to process (n=271) and largely reduced the manual inspection time. This is the reason why we put forward the idea of using the NCBO Annotator to pick out the words related to the biomedical fields.

The ratio of misspelled words, which were appropriately corrected in the 58 matched pairs, was close to the overall spelling errors (the difference was 0.97%, less than 1%), and the accuracy (50/58, 86%) fell within the reliable range, within one standard deviation from the mean value of 85.2% (range 80.6% to 89.9%). For these reasons, the NCBO Annotator can well represent the overall performance of our system.

### Future Work

In future work, different types of ontologies are needed to test and verify whether our evaluation method can be applied in other fields. Moreover, we will add and mix more correction tools in addition to Google Spell Checker to promote the overall performance of our spelling correction system.

### Conclusions

From this study, the following can be confirmed:

1. Our system is suitable for spelling correction in consumer-generated content. The unique features in consumer-generated content have been identified and taken into consideration. Google Spell Checker displays high performance in spelling error detection and correction in consumer-generated content.

2. Context sensitivity is indispensable in the correction process.

3. Our evaluation method, taking advantage of biomedical ontology, can effectively evaluate the correction system and reduce manual inspection time on a large scale.

4. In consumer-generated content, consumers rarely use abbreviations, unlike in EMRs.

## Appendix

Multimedia Appendix 1 Original data: 150 postings.

Multimedia Appendix 2 Result file.

## Abbreviations

API: application programming interface
BBS: bulletin board system
C: corrected
EMR: electronic medical record
gtt: guttae
L: linguistic error
NCBO: National Center for Biomedical Ontology
NLP: natural language processing
NTG: nitroglycerin
PVCs: premature ventricular contractions
SNOMED CT: Systematized Nomenclature of Medicine -- Clinical Terms
T: typographical error
U: uncorrected
VSS: vital signs stable
