# Supplementary material for: Context-Sensitive Spelling Correction of Consumer-Generated Content on Health Care
Source: JMIR Med Inform. 2015 Jul 31;3(3):e27. doi: 10.2196/medinform.4211 (PMC4705358; doi:10.2196/medinform.4211)
Supplement: Multimedia Appendix 2 [file medinform_v3i3e27_app2.pdf]

Supplementary Document

1 Google-Spell-Checker-embedded System

There are 271 spelling errors detected by this system, and these spelling errors are categorized as follows:

Note:

1 "+" means the corresponding error is corrected appropriately, and "-" means it is corrected inappropriately.

*2 "^##^A_c^##^A_un^##^" means the error is a typographical error, and "^**^A_c^**^A_un^**^" means it is a linguistic error. (A_un is the uncorrected word, and A_c is the corrected one)*

1.1 Corrected appropriately (231 spelling errors)

+ ^##^guarantee^##^guarranty^##^
+ ^##^antidepressants.^##^anti-depressants.^##^
+ ^##^Good^##^??Good^##^
+ ^##^antidepressant^##^anti-depressant^##^
+ ^##^antidepressant^##^anti-depressant^##^
+ ^##^us^##^ujs^##^
+ ^##^working.^##^workng.^##^
+ ^##^Definitely^##^Definetly^##^
+ ^##^taper^##^tapered^##^
+ ^##^didn't^##^didnt^##^
+ ^##^Pristiq,^##^Prestiq,^##^
+ ^##^similar^##^similiar^##^
+ ^##^hadn't^##^hadnt^##^
+ ^##^wouldn't^##^wouldnt^##^
+ ^##^antidepressant^##^anti-depressant^##^
+ ^##^Sertraline^##^Setraline^##^
+ ^##^antidepressants.^##^anti-depressants.^##^
+ ^##^prescribed^##^perscribed^##^
+ ^##^approaching^##^aproaching^##^
+ ^##^psychologist^##^spycologist^##^
+ ^##^sole^##^soul^##^

+ ^##^stuff.^##^stuiff.^##^
+ ^##^the^##^ther^##^
+ ^##^separate^##^seperate^##^
+ ^##^concerns^##^concernsm^##^
+ ^##^PTSD^##^PTSS^##^
+ ^##^We^##^????We^##^
+ ^##^I^##^??I^##^
+ ^##^so^##^sooooo^##^
+ ^##^it's^##^??it's^##^
+ ^##^but^##^??but^##^
+ ^##^Thanks^##^????Thanks^##^
+ ^##^wasn't^##^wasn’t^##^
+ ^##^:-/^##^:-/??^##^
+ ^##^I^##^??I^##^
+ ^##^Within^##^??Within^##^
+ ^##^I^##^??I^##^
+ ^##^Lesson^##^??Lesson^##^
+ ^##^initial^##^intital^##^
+ ^##^ridiculously^##^rediculously^##^
+ ^##^Hmmm.^##^Hmmmmm.^##^
+ ^##^Serotonin^##^Seratonin^##^
+ ^##^Norepinephrine.^##^Norapinefrine.^##^
+ ^##^Norepinephrine^##^Norapinefrine^##^
+ ^##^Hmmm.^##^Hmmmmm.^##^
+ ^##^antidepressant^##^anti-depressant^##^
+ ^##^No^##^??No^##^
+ ^##^I^##^??I^##^
+ ^##^adipex^##^arrapax^##^
+ ^##^episodes^##^epsiodes^##^
+ ^##^exercise^##^excersice^##^
+ ^##^don't^##^dont^##^
+ ^##^that^##^thtat^##^
+ ^##^(serotonin^##^(seretonin^##^
+ ^##^psychiatrist^##^prychiatrist^##^
+ ^##^give^##^giv^##^
+ ^##^yourself^##^yoursel^##^
+ ^##^Best^##^??Best^##^

+ ^##^zoloft^##^zolof^##^
+ ^##^sugar.^##^suger.^##^
+ ^##^Zoloft^##^Zolof^##^
+ ^##^taper^##^tapered^##^
+ ^##^concern^##^conern^##^
+ ^##^taper^##^tapered^##^
+ ^##^acupuncture!!^##^accupunture!!^##^
+ ^##^It's^##^??It's^##^
+ ^##^Your^##^??Your^##^
+ ^##^I^##^??I^##^
+ ^##^Check^##^??Check^##^
+ ^##^research^##^reserach^##^
+ ^##^The^##^??The^##^
+ ^##^Eat^##^??Eat^##^
+ ^##^Serotonin^##^??Serotonin^##^
+ ^##^Just^##^??Just^##^
+ ^##^antidepressant--------I^##^anti-depressant--------I^##^
+ ^##^I^##^??I^##^
+ ^##^taper^##^tapper^##^
+ ^##^I^##^??I^##^
+ ^##^It's^##^??It's^##^
+ ^##^But^##^??But^##^
+ ^##^Well.^##^??Well.^##^
+ ^##^I'm^##^??I'm^##^
+ ^##^beginning^##^beggining^##^
+ ^##^I^##^??I^##^
+ ^##^I'm^##^??I'm^##^
+ ^##^exercising^##^excersizing^##^
+ ^##^I'm^##^??I'm^##^
+ ^##^I^##^??I^##^
+ ^##^Will^##^??Will^##^
+ ^##^On^##^??On^##^
+ ^##^I've^##^??I've^##^
+ ^##^Zoloft.^##^Zolof.^##^
+ ^##^I'm^##^??I'm^##^
+ ^##^I^##^??I^##^
+ ^##^6^##^??6^##^

+ ^##^Not^##^??Not^##^
+ ^##^No^##^??No^##^
+ ^##^It^##^??It^##^
+ ^##^I^##^??I^##^
+ ^##^losing^##^loosing^##^
+ ^##^I^##^??I^##^
+ ^##^I^##^??I^##^
+ ^##^Although^##^??Although^##^
+ ^##^Suddenly,^##^??Suddenly,^##^
+ ^##^We'll^##^??We'll^##^
+ ^##^Zyprexa^##^Zyprexia^##^
+ ^##^habit^##^habbit^##^
+ ^##^loss^##^woss^##^
+ ^##^I^##^??I^##^
+ ^##^This^##^??This^##^
+ ^##^terrifies^##^terrifyes^##^
+ ^##^I^##^??I^##^
+ ^##^exercise^##^excersise^##^
+ ^##^I'm^##^??I'm^##^
+ ^##^I've^##^Ive^##^
+ ^##^beginning^##^beggining^##^
+ ^##^appetite^##^appatite^##^
+ ^##^Been^##^??Been^##^
+ ^##^I^##^??I^##^
+ ^##^I^##^??I^##^
+ ^##^losing^##^loosing^##^
+ ^##^weight^##^weigtht^##^
+ ^##^I^##^??I^##^
+ ^##^Exercise^##^??Exercise^##^
+ ^##^good^##^??good^##^
+ ^##^When^##^??When^##^
+ ^##^While^##^??While^##^
+ ^##^It^##^??It^##^
+ ^##^I^##^??I^##^
+ ^##^Good^##^??Good^##^
+ ^##^As^##^??As^##^
+ ^##^Initially,^##^??Initially,^##^

- ^##^After^##^??After^##^
- ^##^I^##^??I^##^
- ^##^After^##^??After^##^
- ^##^I^##^??I^##^
- ^##^this^##^??this^##^
- ^##^i've^##^??i've^##^
- ^##^more^##^??more^##^
- ^##^i'm^##^??i'm^##^
- ^##^but^##^??but^##^
- ^##^i'm^##^??i'm^##^
- ^##^i^##^??i^##^
- ^##^i'm^##^??i'm^##^
- ^##^but^##^??but^##^
- ^##^i'll^##^??i'll^##^
- ^##^posted,^##^psoted,^##^
- ^##^make^##^dmake^##^
- ^##^amitriptyline^##^amitryptiline^##^
- ^##^Please^##^Pleas^##^
- ^##^Please^##^Pleas^##^
- ^##^Give^##^??Giv^##^
- ^##^SSRIs^##^SSRI's^##^
- ^##^you^##^yhou^##^
- ^##^weaning^##^weening^##^
- ^##^pharmaceutical^##^pharmacutical^##^
- ^##^I^##^??I^##^
- ^##^All^##^??All^##^
- ^##^I^##^??I^##^
- ^##^However,^##^??However,^##^
- ^##^My^##^??My^##^
- ^##^My^##^??My^##^
- ^##^I^##^??I^##^
- ^##^don't^##^dont^##^
- ^##^I^##^??I^##^
- ^##^Now^##^??Now^##^
- ^##^Thanks^##^??Thanks^##^
- ^##^generic^##^genereic^##^
- ^##^occasions^##^occassions^##^

+ ^##^committing^##^comitting^##^
+ ^##^initially^##^iniitally^##^
+ ^##^obviously^##^obviosuly^##^
+ ^##^because^##^becuase^##^
+ ^##^true^##^tru^##^
+ ^##^cuz^##^bcuz^##^
+ ^##^first^##^frist^##^
+ ^##^because^##^becuase^##^
+ ^##^exist^##^exsist^##^
+ ^##^vengeance.^##^vengance.^##^
+ ^##^yourself^##^urself^##^
+ ^##^yourself^##^urself^##^
+ ^##^don't^##^dont^##^
+ ^**^change^**^changed^**^
+ ^**^further^**^farther^**^
+ ^**^than^**^then^**^
+ ^**^hopeless^**^helpless^**^
+ ^**^it's^**^its^**^
+ ^**^too^**^to^**^
+ ^**^obtain^**^retain^**^
+ ^**^dr^**^doc^**^
+ ^**^SYS^**^CYS^**^
+ ^**^years^**^yrs^**^
+ ^**^advice^**^advise^**^
+ ^**^today's^**^todays^**^
+ ^**^doctor's^**^doctors^**^
+ ^**^can't^**^cant^**^
+ ^**^hygiene^**^hygeine^**^
+ ^**^EXTRA^**^ULTRA^**^
+ ^**^severe^**^serve^**^
+ ^**^that's^**^thats^**^
+ ^**^its^**^it's^**^
+ ^**^lower^**^lover^**^
+ ^**^and^**^or^**^
+ ^**^weigh^**^weight^**^
+ ^**^You're^**^Your^**^
+ ^**^not^**^now^**^

+ ^**^lose^**^lost^**^
+ ^**^alone!^**^alone!??^**^
+ ^**^lose^**^loose^**^
+ ^**^lose^**^loose^**^
+ ^**^calendar.^**^calender.^**^
+ ^**^lose^**^loose^**^
+ ^**^keep^**^kept^**^
+ ^**^and^**^then^**^
+ ^**^thought,^**^though,^**^
+ ^**^cause^**^caused^**^
+ ^**^thought,^**^though,^**^
+ ^**^lose^**^loose^**^
+ ^**^than^**^then^**^
+ ^**^happened^**^happen^**^
+ ^**^worst^**^worse^**^
+ ^**^wean^**^ween^**^
+ ^**^doctors^**^drs^**^
+ ^**^10^**^10+^**^
+ ^**^into^**^onto^**^
+ ^**^and^**^or^**^
+ ^**^its^**^it's^**^
+ ^**^advice^**^advise^**^
+ ^**^thru^**^threw^**^
+ ^**^taking^**^talking^**^
+ ^**^you^**^u^**^

1.2 Corrected inappropriately (40 spelling errors)

- ^##^are^##^ate^##^
- ^##^effective.^##^ineffective.^##^
- ^##^/-^##^+/-^##^
- ^##^Zolof^##^Zoloft^##^
- ^**^continue^**^continued^**^
- ^**^does^**^dose^**^
- ^**^to,^**^too,^**^
- ^**^packing^**^pacing^**^
- ^**^has^**^had^**^
- ^**^seems^**^seemed^**^
- ^**^quit^**^quite^**^

- ^**^shaking.^**^shaken.^**^
- ^**^does?^**^dose?^**^
- ^**^helpful^**^unhelpful^**^
- ^**^mad^**^med^**^
- ^**^taper^**^tapered^**^
- ^**^the^**^then^**^
- ^**^your^**^our^**^
- ^**^choose^**^chose^**^
- ^**^withdraw,^**^withdrawal,^**^
- ^**^our^**^your^**^
- ^**^withdraw^**^withdrew^**^
- ^**^your^**^our^**^
- ^**^your^**^our^**^
- ^**^poops^**^pooped^**^
- ^**^cause^**^cuased^**^
- ^**^carbs,^**^herbs,^**^
- ^**^pulse^**^impulse^**^
- ^**^keep^**^kept^**^
- ^**^fingered^**^figured^**^
- ^**^through,^**^though,^**^
- ^**^lose^**^lost^**^
- ^**^a^**^u^**^
- ^**^e^**^a^**^
- ^**^alleviate^**^alleviated^**^
- ^**^of^**^or^**^
- ^**^through^**^though^**^
- ^**^Set^**^Sert^**^
- ^**^Chemistry,^**^Chemist,^**^
- ^**^wake^**^woke^**^

2 Classification of typographical and linguistic errors

2.1 Typographical errors (186 spelling errors)

+ ^##^guarantee^##^guarranty^##^
+ ^##^antidepressants.^##^anti-depressants.^##^
+ ^##^Good^##^??Good^##^
+ ^##^antidepressant^##^anti-depressant^##^

+ ^##^antidepressant^##^anti-depressant^##^
+ ^##^us^##^ujs^##^
+ ^##^working.^##^workng.^##^
+ ^##^Definitely^##^Definetly^##^
+ ^##^taper^##^tapered^##^
+ ^##^didn't^##^didnt^##^
+ ^##^Pristiq,^##^Prestiq,^##^
+ ^##^similar^##^similiar^##^
+ ^##^hadn't^##^hadnt^##^
+ ^##^wouldn't^##^wouldnt^##^
+ ^##^antidepressant^##^anti-depressant^##^
+ ^##^Sertraline^##^Setraline^##^
+ ^##^antidepressants.^##^anti-depressants.^##^
+ ^##^prescribed^##^perscribed^##^
+ ^##^approaching^##^aproaching^##^
+ ^##^psychologist^##^spycologist^##^
+ ^##^sole^##^soul^##^
+ ^##^stuff.^##^stuiff.^##^
+ ^##^the^##^ther^##^
+ ^##^separate^##^seperate^##^
+ ^##^concerns^##^concernsm^##^
+ ^##^PTSD^##^PTSS^##^
+ ^##^We^##^????We^##^
+ ^##^I^##^??I^##^
+ ^##^so^##^sooooo^##^
+ ^##^it's^##^??it's^##^
+ ^##^but^##^??but^##^
+ ^##^Thanks^##^????Thanks^##^
+ ^##^wasn't^##^wasn’t^##^
+ ^##^:-/^##^:-/??^##^
+ ^##^I^##^??I^##^
+ ^##^Within^##^??Within^##^
+ ^##^I^##^??I^##^
+ ^##^Lesson^##^??Lesson^##^
+ ^##^initial^##^intital^##^
+ ^##^ridiculously^##^rediculously^##^
+ ^##^Hmmm.^##^Hmmmmm.^##^

+ ^##^Serotonin^##^Seratonin^##^
+ ^##^Norepinephrine.^##^Norapinefrine.^##^
+ ^##^Norepinephrine^##^Norapinefrine^##^
+ ^##^Hmmm.^##^Hmmmmm.^##^
+ ^##^antidepressant^##^anti-depressant^##^
+ ^##^No^##^??No^##^
+ ^##^I^##^??I^##^
+ ^##^adipex^##^arrapax^##^
+ ^##^episodes^##^epsiodes^##^
+ ^##^exercise^##^excersice^##^
+ ^##^don't^##^dont^##^
+ ^##^that^##^thtat^##^
+ ^##^(serotonin^##^(seretonin^##^
+ ^##^psychiatrist^##^prychiatrist^##^
+ ^##^give^##^giv^##^
+ ^##^yourself^##^yoursel^##^
+ ^##^Best^##^??Best^##^
+ ^##^zoloft^##^zolof^##^
+ ^##^sugar.^##^suger.^##^
+ ^##^Zoloft^##^Zolof^##^
+ ^##^taper^##^tapered^##^
+ ^##^concern^##^conern^##^
- ^##^are^##^ate^##^
+ ^##^taper^##^tapered^##^
+ ^##^acupuncture!!^##^accupunture!!^##^
- ^##^effective.^##^ineffective.^##^
+ ^##^It's^##^??It's^##^
+ ^##^Your^##^??Your^##^
+ ^##^I^##^??I^##^
+ ^##^Check^##^??Check^##^
+ ^##^research^##^reserach^##^
+ ^##^The^##^??The^##^
+ ^##^Eat^##^??Eat^##^
+ ^##^Serotonin^##^??Serotonin^##^
+ ^##^Just^##^??Just^##^
+ ^##^antidepressant--------I^##^anti-depressant--------I^##^
+ ^##^I^##^??I^##^

+ ^##^taper^##^tapper^##^
+^##^I^##^??I^##^
+^##^It's^##^??It's^##^
+^##^But^##^??But^##^
+^##^Well.^##^??Well.^##^
+^##^I'm^##^??I'm^##^
+^##^beginning^##^beggining^##^
+^##^I^##^??I^##^
+^##^I'm^##^??I'm^##^
+^##^exercising^##^excersizing^##^
+^##^I'm^##^??I'm^##^
+^##^I^##^??I^##^
+^##^Will^##^??Will^##^
+^##^On^##^??On^##^
+^##^I've^##^??I've^##^
+^##^Zoloft.^##^Zolof.^##^
+^##^I'm^##^??I'm^##^
+^##^I^##^??I^##^
+^##^6^##^??6^##^
+^##^Not^##^??Not^##^
+^##^No^##^??No^##^
+^##^It^##^??It^##^
+^##^I^##^??I^##^
+^##^losing^##^loosing^##^
+^##^I^##^??I^##^
+^##^I^##^??I^##^
+^##^Although^##^??Although^##^
+^##^Suddenly,^##^??Suddenly,^##^
+^##^We'll^##^??We'll^##^
+^##^Zyprexa^##^Zyprexia^##^
+^##^habit^##^habbit^##^
+^##^loss^##^woss^##^
+^##^I^##^??I^##^
+^##^This^##^??This^##^
+^##^terrifies^##^terrifyes^##^
+^##^I^##^??I^##^
+^##^exercise^##^excersise^##^

+^##^I'm^##^??I'm^##^

+^##^I've^##^Ive^##^

+^##^beginning^##^beggining^##^

+^##^appetite^##^appatite^##^

+^##^Been^##^??Been^##^

+^##^I^##^??I^##^

+^##^I^##^??I^##^

+^##^losing^##^loosing^##^

+^##^weight^##^weigtht^##^

+^##^I^##^??I^##^

+^##^Exercise^##^??Exercise^##^

+^##^good^##^??good^##^

+^##^When^##^??When^##^

+^##^While^##^??While^##^

+^##^It^##^??It^##^

+^##^I^##^??I^##^

+^##^Good^##^??Good^##^

+^##^As^##^??As^##^

+^##^Initially,^##^??Initially,^##^

+^##^After^##^??After^##^

+^##^I^##^??I^##^

+^##^After^##^??After^##^

+^##^I^##^??I^##^

+^##^this^##^??this^##^

+^##^i've^##^??i've^##^

+^##^more^##^??more^##^

+^##^i'm^##^??i'm^##^

+^##^but^##^??but^##^

+^##^i'm^##^??i'm^##^

+^##^i^##^??i^##^

+^##^i'm^##^??i'm^##^

+^##^but^##^??but^##^

+^##^i'll^##^??i'll^##^

+^##^posted,^##^psoted,^##^

+^##^make^##^dmake^##^

+^##^amitriptyline^##^amitryptiline^##^

+^##^Please^##^Pleas^##^

+^##^Please^##^Pleas^##^

+^##^Give^##^??Giv^##^

+^##^SSRIs^##^SSRI's^##^

+^##^you^##^yhou^##^

+^##^weaning^##^weening^##^

+^##^pharmaceutical^##^pharmacutical^##^

+^##^I^##^??I^##^

+^##^All^##^??All^##^

+^##^I^##^??I^##^

+^##^However,^##^??However,^##^

-^##^/-^##^+/-^##^

+^##^My^##^??My^##^

+^##^My^##^??My^##^

+^##^I^##^??I^##^

+^##^don't^##^dont^##^

+^##^I^##^??I^##^

+^##^Now^##^??Now^##^

+^##^Thanks^##^??Thanks^##^

+^##^generic^##^genereic^##^

+^##^occasions^##^occassions^##^

+^##^committing^##^comitting^##^

-^##^Zolof^##^Zoloft^##^

+^##^initially^##^iniitally^##^

+^##^obviously^##^obviosuly^##^

+^##^because^##^becuase^##^

+^##^true^##^tru^##^

+^##^cuz^##^bcuz^##^

+^##^first^##^frist^##^

+^##^because^##^becuase^##^

+^##^exist^##^exsist^##^

+^##^vengeance.^##^vengance.^##^

+^##^yourself^##^urself^##^

+^##^yourself^##^urself^##^

+^##^don't^##^dont^##^

2.2 Linguistic errors (85 spelling errors)

+ ^**^change^**^changed^**^

- ^**^continue^**^continued^**^

- ^**^does^**^dose^**^
- ^**^to,^**^too,^**^
+ ^**^further^**^farther^**^
- ^**^packing^**^pacing^**^
- ^**^has^**^had^**^
- ^**^seems^**^seemed^**^
- ^**^quit^**^quite^**^
- ^**^shaking.^**^shaken.^**^
- ^**^does?^**^dose?^**^
+ ^**^than^**^then^**^
- ^**^helpful^**^unhelpful^**^
+ ^**^hopeless^**^helpless^**^
+ ^**^it's^**^its^**^
+ ^**^too^**^to^**^
+ ^**^obtain^**^retain^**^
- ^**^mad^**^med^**^
+ ^**^dr^**^doc^**^
- ^**^taper^**^tapered^**^
- ^**^the^**^then^**^
+ ^**^SYS^**^CYS^**^
- ^**^your^**^our^**^
+ ^**^years^**^yrs^**^
- ^**^choose^**^chose^**^
+ ^**^advice^**^advise^**^
+ ^**^today's^**^todays^**^
- ^**^withdraw,^**^withdrawal,^**^
- ^**^our^**^your^**^
+ ^**^doctor's^**^doctors^**^
+ ^**^can't^**^cant^**^
- ^**^withdraw^**^withdrew^**^
+ ^**^hygiene^**^hygeine^**^
+ ^**^EXTRA^**^ULTRA^**^
- ^**^your^**^our^**^
- ^**^your^**^our^**^
- ^**^poops^**^pooped^**^
+ ^**^severe^**^serve^**^
+ ^**^that's^**^thats^**^

+ ^**^its^**^it's^**^
+ ^**^lower^**^lover^**^
+ ^**^and^**^or^**^
- ^**^cause^**^cuased^**^
- ^**^carbs,^**^herbs,^**^
+ ^**^weigh^**^weight^**^
+ ^**^You're^**^Your^**^
+ ^**^not^**^now^**^
+ ^**^lose^**^lost^**^
+ ^**^alone!^**^alone!??^**^
+ ^**^lose^**^loose^**^
+ ^**^lose^**^loose^**^
+ ^**^calendar.^**^calender.^**^
+ ^**^lose^**^loose^**^
- ^**^pulse^**^impulse^**^
- ^**^keep^**^kept^**^
+ ^**^keep^**^kept^**^
+ ^**^and^**^then^**^
+ ^**^thought,^**^though,^**^
+ ^**^cause^**^caused^**^
- ^**^fingered^**^figured^**^
- ^**^through,^**^though,^**^
+ ^**^thought,^**^though,^**^
+ ^**^lose^**^loose^**^
+ ^**^than^**^then^**^
+ ^**^happened^**^happen^**^
+ ^**^worst^**^worse^**^
- ^**^lose^**^lost^**^
+ ^**^wean^**^ween^**^
+ ^**^doctors^**^drs^**^
- ^**^a^**^u^**^
- ^**^e^**^a^**^
- ^**^alleviate^**^alleviated^**^
+ ^**^10^**^10+^**^
- ^**^of^**^or^**^
- ^**^through^**^though^**^
+ ^**^into^**^onto^**^

- ^**^Set^**^Sert^**^
+ ^**^and^**^or^**^
- ^**^Chemistry,^**^Chemist,^**^
+ ^**^its^**^it's^**^
+ ^**^advice^**^advise^**^
- ^**^wake^**^woke^**^
+ ^**^thru^**^threw^**^
+ ^**^taking^**^talking^**^
+ ^**^you^**^u^**^

3 Spelling pairs matched in ontology (58 spelling pairs)

3.1 Corrected appropriately (50 spelling pairs)

+ ^##^Good^##^??Good^##^
+ ^##^antidepressant^##^anti-depressant^##^
+ ^##^antidepressant^##^anti-depressant^##^
+ ^##^antidepressant^##^anti-depressant^##^
+ ^##^antidepressant^##^anti-depressant^##^
+ ^##^antidepressant--------I^##^anti-depressant--------I^##^
+ ^##^working.^##^workng.^##^
+ ^##^Pristiq,^##^Prestiq,^##^
+ ^##^Sertraline^##^Setraline^##^
+ ^##^psychologist^##^spycologist^##^
+ ^##^separate^##^seperate^##^
+ ^##^initial^##^intital^##^
+ ^##^Serotonin^##^Seratonin^##^
+ ^##^(serotonin^##^(seretonin^##^
+ ^##^Serotonin^##^??Serotonin^##^
+ ^##^Norepinephrine.^##^Norapinefrine.^##^
+ ^##^Norepinephrine^##^Norapinefrine^##^
+ ^##^episodes^##^epsiodes^##^
+ ^##^exercise^##^excersice^##^
+ ^##^psychiatrist^##^prychiatrist^##^
+ ^##^zoloft^##^zolof^##^
+ ^##^Zoloft^##^Zolof^##^
+ ^##^sugar.^##^suger.^##^
+ ^##^acupuncture!!^##^accupunture!!^##^
+ ^##^Zoloft.^##^Zolof.^##^

+ ^##^Zyprexa^##^Zyprexia^##^
+ ^##^habit^##^habbit^##^
+ ^##^exercise^##^excersise^##^
+ ^##^Exercise^##^??Exercise^##^
+ ^##^appetite^##^appatite^##^
+ ^##^good^##^??good^##^
+ ^##^Good^##^??Good^##^
+ ^##^initially^##^iniitally^##^
+ ^##^Initially,^##^??Initially,^##^
+ ^##^amitriptyline^##^amitryptiline^##^
+ ^##^weaning^##^weening^##^
+ ^##^true^##^tru^##^
+ ^**^change^**^changed^**^
+ ^**^dr^**^doc^**^
+ ^**^years^**^yrs^**^
+ ^**^today's^**^todays^**^
+ ^**^doctor's^**^doctors^**^
+ ^**^severe^**^serve^**^
+ ^**^lower^**^lover^**^
+ ^**^lose^**^loose^**^
+ ^**^lose^**^loose^**^
+ ^**^lose^**^loose^**^
+ ^**^lose^**^loose^**^
+ ^**^worst^**^worse^**^
+ ^**^taking^**^talking^**^

3.2 Corrected inappropriately (8 spelling pairs)

- ^##^effective.^##^ineffective.^##^
- ^##^Zolof^##^Zoloft^##^
- ^**^does^**^dose^**^
- ^**^does?^**^dose?^**^
- ^**^packing^**^pacing^**^
- ^**^shaking.^**^shaken.^**^
- ^**^pulse^**^impulse^**^
- ^**^Chemistry,^**^Chemist,^**^

4 Abbreviations in the postings

a.m	mg	SSRI	mg	OCD	mg	meds	Rx’d	ujs	mg	Rx	Rx	OB	meds
meds	mg	mg	mg	OMG	med	drs	dr	mg	dr	meds	meds	dr	meds
med	meds	mg	mgs	ex	ex	ex	PTSS	yrs	P.S.	mg	mg	SSRI	SSRIs
TV	FDA	XR	BTW	med	hon	Dr.	RN	Amd	mg	bs	meds	meds	meds
mg	mf	mf	P.S.	idk	u	etc	info	mg	lbs	mos	meds	w/o	MIT
med	etc	med	lbs	meds	mg	b/c	mg	lbs	mg	lbs	lbs	SSRI	lbs
lbs	lbs	lbs	etc	mg	lbs	med	lbs	lbs	mg	mg	lbs	Dr	oz
org	lbs	mg	mg	mg	AM	hr	hr	lbs	EFX	mg	OCD	lbs	mg
mg	lbs	etc	hr	lbs	lbs	lbs	lbs	lbs	lbs	lbs	lb	lbs	mg
mg	mg	vs.	mg	lbs	lbs	M4YOU	lbs	SSRIs	lbs	lbs	PUI	yrs	Lo
mg	PTSD	lbs	lbs	lbs	mg	lbs	mg	mg	yrs	meds	u	r	u
r	u	r	u	r	u	u	u	r	u	u	u	u	u
meds	SSRI	OCD	BK	mg	meds	mg	mg	mg	yrs	vs	2nd	6th	17th
5th	etc.	dr	FDA	FDA	mg	2nd	3rd	dr	mg	bpm	PCP	meds	meds
2morrow	ya	meds	mg	PSTD	mg	mg	SSRIs	mg	meds	mg	mg	u	i.e

5 Microsoft Word 2013 Spell Checker

Note: the result is presented in the form “after-correction / before-correction”, and if no spelling suggestion is provided from this system, the after-correction

part will be left in blank.

5.1 Corrected appropriately (313 spelling errors)

self-confidence / self confidence
self-worth / self worth
guaranty / guarranty
ha-ha. / haha
Zoloft / zoloft
There / there
working / workng
Zoloft / zoloft
ha-ha. / haha
a while / awhile
Like / like
ha-ha. / haha
a while / awhile
Like / like
Nurse Girl / Nursegirl
I’ll / i'll
Zoloft / zoloft
won’t / wont
self-fulfilling / self fulfilling
Zoloft / zoloft
what / What
Definitely / Definetly
Zoloft / zoloft
didn’t / didnt
couldn’t / couldnt
any more / anymore
Effexor / effexor
any more / anymore
similar / similiar
a lot / alot
hadn’t / hadnt
wouldn’t / wouldnt
Sertraline / Setraline
though / tho

don’t / dont
though / tho
prescribed / perscribed
approaching / aproaching
psychologist / spycologist
stuff / stuiff
the / ther
separate / seperate
concerns / concernsm
non-judgmental / non-judgemental
self-esteem / self esteem
emerge / emerg
Even / even
It’s / it's
But / but
wasn’t / wasn¡¯t
Paxil / paxil
Lisinopril / lisinopril
anti-depressants / anti depressants
initial / ntital
hygiene / hygeine
ridiculously / rediculously
Once / once
Effexor / effexor
Serotonin / Seratonin
Norepinephrine / Norapinefrine
Norepinephrine / Norapinefrine
it’s / its
for panic / for panic
cannot / can not
given? / given.
home. / home .
going to / gonna
well / better
what my / what my
it’s / its
it’s / its

time. Good / time.Good
on many / on many
it’s / its
may be / maybe
ex-suffer / ex suffer
In / in
breakdown / break down
advice / advise
today’s / todays
concern. / concern?
doctor’s / doctors
can’t / cant
known / know
Mother’s Day / Mothers Day
hon / hone
effexor / Effexor
know / known
And / Amd
Effexor / effexor
Zoloft / zoloft
Paxil / paxil
worthwhile / worth while
Effexor / effexor
anti-depressants / anti depressants
anti-depressants / anti depressants
Zoloft / zoloft
episodes / epsiodes
irk / idk
don’t / dont
that’s / thats
is / i
is / i
exercise / excersice
Your / YOur
fine!! / fine !!
Really / really
don’t / dont

that / thtat
you. / you .
with / With
serotonin / seretonin
too / to
that’s / thats
psychiatrist / prychiatrist
give / giv
yoursel / yourself
Zoloft / zoloft
Zoloft / zolof
exercise, and / exercise,and
Zoloft / Zolof
concern / conern
sugar / suger
Loser? / Loser.
lbs. / lbs,
mos. / mos
Zoloft / zoloft
losing / loosing
acupuncture / accupunture
cuased / cuased
Zoloft / zoloft
Zoloft / zoloft
research / reserach
meds? / meds.
Zoloft / zoloft
For / for
you’ve / you’ ve
You’re / Your
Zoloft / zoloft
taper / tapper
lbs. / lbs
weight.” / weight ."
Zoloft / zoloft
Zoloft / zoloft
beginning / beggining

Zoloft / zoloft
lbs. / lbs
Zoloft / zoloft
lose / loose
losing / loosing
lbs. / lbs
exercising / excersizing
Zoloft / Zolof
Zoloft / Zolof
Zoloft / zoloft
and since / and since
lbs. / lbs
Dry’s / Dr's
Zoloft / zoloft
losing / loosing
lose / loose
lose / loose
Zoloft / zoloft
lbs. / lbs
weight loss / weightloss
yrs. / yrs
Zoloft / zoloft
Zoloft / zoloft
and then / then
Zyprexa / Zyprexia
calendar / calender
lose / loose
lose / loose
habit / habbit
Zoloft / zoloft
terrifies / terrifyes
Zoloft / zoloft
hr. / hr
Counting / counting
apt / appt
Zoloft / zoloft
Zoloft / zoloft

Zoloft / zoloft
Zoloft / zoloft
Zoloft / zoloft
lbs. / lbs
Zoloft / zoloft
excercise / excersise
, no / , no
lbs. / lbs
I’ve / i've
I’ve / Ive
beginning / beggining
pounds / ponds
appetite / appatite
a lot / alot
every day / everyday
losing / loosing
weight / weigtht
doing / doind
lbs. / lbs
lbs. / lbs
yrs. / yrs
Zoloft / zoloft
Neurontin / neurontin
cannot / can not
Zoloft / zoloft
lose / loose
every day / everyday
than / then
won’t / wont
lbs. / lbs
lbs. / lbs
wean / ween
Zoloft / zoloft
Zoloft / zoloft
Zoloft / zoloft
than / then
gain? / gain.

posted / psoted
week? / week.
than / then
chose to / chose to
make / dmake
self-help / self help
self-esteem / self esteem
Try / try
amitriptyline / amitryptiline
a while / awhile
Please / Pleas
Prozac / prozac
Prozac / prozac
Effexor / effexor
daily / dailyh
Prozac / prozac
Jessica / jessica
Zoloft / zoloft
Zoloft / zoloft
Zoloft / zoloft
weaning / weening
Zoloft / zoloft
yrs. / yrs
pharmaceutical / pharmacutical
my pharmacist / my pharmacist
the / they
and dizziness / dizziness
Zoloft / zoloft
don't / dont
generic / genereic
With the / With the
occasions / occassions
committing / comitting
Approx. / Approx
its / it's
advice / advise
needs? / needs.

initally / iniitally
heartbeat / heart beat
affect / effect
obviously / obviosuly
in case / incase
hear / here
different / diffrent
stories / storys
Zoloft / zoloft
dying / diein
everywhere / every where
joint pain / jointpain
nausea / nauses
chores / choures
I’ve / ive
myself / my self
synthoms / synthoms
because / sbecuase
don't / dont
through / threw
hyperthyroidism / hiperthyriodism
any moment / anymoment
knuckles / nuckles
first / frist
because / becuase
because / becuase
doesn't / doesnt
weird / wierd
different / diffrent
don’t / dont
past / pass
suicidal / sucidal
that's / thats
possessed / possesed
true / tru
what's / whats
because / becuase

Zoloft / zoloft
overwhelming / overwellming
Zoloft / zoloft
overwhelming / overwellming
didn't / didnt
suicidal / sucidal
walking / walkin
I was / iwas
several / severl
counldn't / counldnt
hands / hads
because / becuase
exists / exsist
diarrhea / diarrhoea
vengeance / vengance
, I / , I
self-esteem / self esteem
yourself. / yourself .
than / then
don't / dont

5.2 Corrected inappropriately (118 spelling errors)

Wilburton / Wellbutrin
thiamine / theanine
Worth / Wort
thiamine / theanine
Rd. / Rx'd
us / ujs
Celera / Celexa
Celera / Celexa
Celera / Celexa
Celera / Celexa
Celera / Celexa
Celera / Celexa
Celera / Celexa
Celera / Celexa
thiamine / theanine
thiamine / theanine

thiamine / theanine
hyping / hypnic
hyping / hypnic
hyping / hypnic
Presto / Prestiq
dress / drs
Presto / Prestiq
Presto / Prestiq
dress / drs
solo / sooooo
mamma’s / mammo's
Wilburton / Wellbutrin
loll / lol
Wilburton / Wellbutrin
Wilburton / Wellbutrin
Wilburton / Wellbutrin
media / meds.I
Dry / Dr
Dry / Dr
Dry / Dr
Dry / Dr
cut / cuz
Dry / Dr
Dry / Dr
Dry / Dr
too / to
menthe / me.The
Carol Ann’s / CarolAnn's
Carol Ann / CarolAnn
once / Once
cling / c'ing
arrayal / arrapax
She / she
She / she
trip tans / triptans
El-Dave / el-dave
woos / woss

mins / mines
dry / dr
Bus par / Buspar
is / i
is / i
milt / milti
 / prothiaden
years’ / years.
months months,I
it / it,I
is / i
nick nyc
is / i
is / i
is / i
is / i
It / it
its / it's
wellborn / wellbutrin
bet / be
form / frm
is / i
be / bk
be / bk
antidepressants? / antidepressants.
is / i
Sort / Sert
Lupine / Lupin
Tea / Teva
Tea / Teva
Tea / Teva
Tea / Teva
Tea / Teva
Tea / Teva
Tea / Teva
Tea / Teva
Sterna / Setrona

Sterna / Setrona
Sterna / Setrona
Sterna / Setrona
dry / dr
is / i
is / i
is / i
is / i
is / i
is / i
is / i
is / i
is / i
is / i
Celera / celexa
is / i
is / i
is / i
is / i
is / i
is / i
add / adhd
is / i
six / siq
spouse / sopouse
is / i
itself / urself
itself / urself

6 Aspell Spell Checker with general dictionary

Note: the result is presented in the form "after-correction / before-correction", and if no spelling suggestion is provided from this system, the after-correction part will be left in blank.

6.1 Corrected appropriately (304 spelling errors)

guaranty / guarranty
Zoloft / zoloft
working / workng
Zoloft / zoloft
I'll / i'll
Zoloft / zoloft
Zoloft / zoloft
Definitely / Definetly
Zoloft / zoloft
didn't / didnt
Dr / dr
Dr / dr
couldn't / couldnt
Dr / dr
similar / similiiar
Dr / dr
Dr / dr
hadn't / hadnt
wouldn't / wouldnt
prescribed / perscribed
Dr / dr
approaching / aproaching
psychologist / spycologist
old's / olds
stiff / stuiff
separate / seperate
concerns / concernsm
wan / wasn
hygiene / hygeine
ridiculously / rediculously
Serotonin / Seratonin
Dopamine / dopamine
Zoloft / zoloft
apex / arrapax
Zoloft / zoloft
episodes / epsiodes

Your / YOur
that / thtat
Serotonin / Seratonin
psychiatrist / prychiatrist
give / giv
yourself / yoursel
Zoloft / zoloft
Zoloft / zolof
Zoloft / zolof
concern / conern
Zoloft / zoloft
acupuncture / accupunture
caused / cuased
Zoloft / zoloft
Zoloft / zoloft
research / reserach
Zoloft / zoloft
Zoloft / zoloft
Zoloft / zoloft
Zoloft / zoloft
beginning / beggining
Zoloft / zoloft
Zoloft / zoloft
exercising / excersizing
Zoloft / Zolof
Zoloft / Zolof
Zoloft / zoloft
Zoloft / zoloft
Zoloft / zoloft
weight loss / weightloss
Zoloft / zoloft
Zoloft / zoloft
habit / habbit
Zoloft / zoloft
Zoloft / zoloft
Zoloft / zoloft
Zoloft / zoloft

Zoloft / zoloft
Zoloft / zoloft
Zoloft / zoloft
Zoloft / zoloft
excesses1 / excersise
I've / i've
I've / Ive
beginning / beggining
Dr / dr
Zoloft / zoloft
Zoloft / zoloft
weight / weightt
Zoloft / zoloft
Zoloft / zoloft
Zoloft / zoloft
Zoloft / zoloft
I've / i've
Zoloft / zoloft
I'm / i'm
I'm / i'm
I'm / i'm
Zoloft / zoloft
I'll / i'll
Zoloft / zoloft
posted / psoted
from / frm
Prozac / prozac
Prozac / prozac
I'm / i'm
daily / dailyh
Prozac / prozac
little / littlw
thou / yhou
Jessica / jessica
Zoloft / zoloft
Zoloft / zoloft
Zoloft / zoloft

Zoloft / zoloft
Zoloft / zoloft
pharmaceutical / pharmacutical
Zoloft / zoloft
Dr / dr
generic / genereic
occasions / occassions
committing / comitting
Dr / dr
Zoloft / zoloft
initially / iniitally
obviously / obviosuly
Zoloft / zoloft
die in / diein
joint pain / jointpain
anuses / nauses
chores / choures
because / because
hyperthyroidism / hiperthyriodism
because / becuase
because / becuase
doesn't / doesnt
neutrino / neoutrin
weird / wierd
Prozac / prozac
suicidal / sucidal
true / tru
Ur / ur
Zoloft / zoloft
because / becuase
COD / ocd
overwhelming / overwellming
didn't / didnt
suicidal / sucidal
didn't / didnt
spouse / sopouse
serveral / severl

couldn't / couldnt
exist / exsist
Zoloft / zoloft
Zoloft / zoloft
because / becuase
Zoloft / zoloft
Zoloft / zoloft
vengeance / vengance
Zoloft / zoloft
don't / dont
hat's / thats
hat's / thats
hat's / thats
apt / appt
in case / incase
any moment / anymoment
that's / thats
walk in / walkin
Zoloft / zoloft
Zoloft / zoloft
episodes / epsiodes
that's / thats
that's / thats
health care / healthcare
serotonin / seretonin
that's / thats
psychiatrist / prychiatrist
give / giv
yourself / yoursel
Zoloft / zoloft
Zoloft / zoloft
Zoloft / Zoloft
concern / conern
Zoloft / zoloft
acupuncture / accupunture
caused / cuased
Zoloft / zoloft

Zoloft / zoloft
research / reserach
Zoloft / zoloft
Zoloft / zoloft
Zoloft / zoloft
Zoloft / zoloft
beginning / beggining
Zoloft / zoloft
Zoloft / zoloft
exercising / excersizing
Zoloft / Zolof
Zoloft / Zolof
Zoloft / zoloft
Zoloft / zoloft
Zoloft / zoloft
weight loss / weightloss
Zoloft / zoloft
Zoloft / zoloft
habit / habbit
Zoloft / zoloft
Zoloft / zoloft
Dr / dr
Zoloft / zoloft
Zoloft / zoloft
Zoloft / zoloft
Zoloft / zoloft
Zoloft / zoloft
Zoloft / zoloft
I've / i've
beginning / beggining
Zoloft / zoloft
Zoloft / zoloft
weight / weigtht
Zoloft / zoloft
Zoloft / zoloft
change / changed
Zoloft / zoloft

Zoloft / zoloft
I've / i've
Zoloft / zoloft
I'm / i'm
I'm / i'm
I'm / i'm
Zoloft / zoloft
I'll / i'll
Zoloft / zoloft
posted / psoted
make / dmake
Give / Giv
Prozac / prozac
Prozac / prozac
I'm / i'm
daily / dailyh
Prozac / prozac
little / littlw
Jessica / jessica
Zoloft / zoloft
Zoloft / zoloft
Zoloft / zoloft
Zoloft / zoloft
Zoloft / zoloft
pharmaceutical / pharmacutical
Zoloft / zoloft
Lu pin / Lupin
Dr / dr
don't / dont
generic / genereic
occasions / occassions
committing / comitting
Dr / dr
Zoloft / zoloft
initially / iniitally
obviously / obviosuly
in case / incase

different / diffrent
storys / stories
Zoloft / zoloft
dieing / diein
joint pain / jointpain
anuses / nauses
chores / choures
I've / ive
because / becuase
don't / dont
hyperthyroidism / hiperthyroidism
any moment / anymoment
knuckles / nuckles
because / becuase
because / becuase
I've / ive
Prozac / prozac
neutrino / neoutrin
weird / wierd
different / diffrent
don't / dont
suicidal / sucidal
possessed / tposseded
tru / true
Zoloft / zoloft
because / becuase
overwhelming / overwellming
didn't / didnt
didn't / didnt
suicidal / sucidal
walk in / walkin
Monday / monday
suppose / sopouse
several / serverl
couldn't / counldnt
hands / hads
because / becuase

exist / exsist
Zoloft / zoloft
Zoloft / zoloft
Zoloft / zoloft
vengeance / vengance
Zoloft / zoloft
yourself / urself
yourself / urself
don't / dont

6.2 Corrected inappropriately (459 spelling errors)

dint / dont
thee / ther
to days / todays
Epinephrine / Norapinefrine
Epinephrine / Norapinefrine
dint / dont
excessive / excersice
Pyrex / Zyprexia
woes / woss
apatite / appatite
dint / dont
diff rent / diffrent
Ive / ive
dint / dont
frost / frist
Ive / ive
bu spur / buspur
diff rent / diffrent
dint / dont
frost / frist
Iowas / i was
hades / hads
itself / urself
itself / urself
dint / dont
Streamline / Sertraline
melds / meds

Weltering / Wellbutrin
SS RI / SSRI
melds / meds
COD / OCD
Jeanine / theanine
melds / meds
Jeanine / theanine
ha ha / haha
melds / meds
BOD / BPD
melds / meds
RD / Rx'd
US / ujs
Celebs / Celexa
Celebs / Celexa
Celebs / Celexa
Celebs / Celexa
Celebs / Celexa
Celebs / Celexa
Celebs / Celexa
melds / meds
melds / meds
melds / meds
melds / meds
melds / meds
Celebs / Celexa
melds / meds
Jeanine / theanine
melds / meds
over analyzing / overanalyzing
ha ha / haha
Jeanine / theanine
melds / meds
Nurse girl / Nursegirl
2ND / 2nd
cyclones / myoclonus
hyping / hypnic

hyping / hypnic
hyping / hypnic
melds / meds
catastrophes / catastrophize
MG / OMG
Streamline / Sertraline
Prestige / Prestiq
drys / drs
Prestige / Prestiq
Prestige / Prestiq
effect / effexor
alto / alot
melds / meds
CZ / cuz
melds / meds
melds / meds
Streamline / Sertraline
effect / effexor
effect / effexor
Streamline / Sertraline
drys / drs
melds / meds
imperial / omeprazole
Mg's / mgs
Melds / Meds
CYST / CYS
judge mental / judgememtal
PT SS / PTSS
em erg / emerg
SO / sooooo
of fence / offence
SS RI / SSRI
melds / meds
Saris / SSRIs
Lent / learnt
effect / effexor
pixel / paxil

undermine / phentermine
snappily / lisinopril
effect / effexor
undermine / phentermine
undermine / phentermine
undermine / phentermine
undermine / phentermine
undermine / phentermine
crab / carb
undermine / phentermine
undermine / phentermine
mamma's / mammo's
melds / meds
effect / effexor
int ital / intital
effect / effexor
effect / effexor
effect / effexor
Weltering / Wellbutrin
effect / effexor
effect / effexor
Lola / lol
effect / effexor
ZR / XR
Weltering / Wellbutrin
Lola / lol
effect / effexor
effect / effexor
Homonym / Hmmmmm
effect / effexor
Weltering / Wellbutrin
effect / effexor
effect / effexor
Weltering / Wellbutrin
Carol Ann's / CarolAnn's
Carol Ann / CarolAnn
effect / effexor

effect / effexor
Homonym / Hmmmmm
effect / effexor
melds / meds
AMD / Amd
cine / c'ing
effect / effexor
verse / versa
effect / effexor
effect / effexor
effect / effexor
effect / effexor
effect / effexor
effect / effexor
effect / effexor
melds / meds
effect / effexor
effect / effexor
pixel / paxil
melds / meds
crab / carb
effect / effexor
effect / effexor
effect / effexor
ID / idk
Streamline / Sertraline
trip tans / triptans
Streamline / Sertraline
healthcare / healthcare
sager / suger
melds / meds
effect / effexor
melds / meds
melds / meds
melds / meds
melds / meds
vie / ve

Pixel / Paxil
melds / meds
Th's / th's
Expire / Lexapro
Expire / Lexapro
Saris / SSRIs
Eli / el
Dave / dave
Dry's / Dr's
melds / meds
pare / pre
melds / meds
ca lender / calender
crab / carb
terrify es / terrifyes
Min's / mins
EX / EFX
COD / OCD
Buspar / Buspar
Indra / Inderal
Hmm / Hmmm
effect / effexor
effect / effexor
effect / effexor
effect / effexor
effect / effexor
alto / alot
mil ti / milti
effect / effexor
protean / prothiaden
do ind / doind
Saris / SSRIs
neuron tin / neurontin
NYC / nyc
PT SD / PTSD
drys / drs
Weltering / Wellbutrin

melds / meds
melds / meds
damage / dmake
melds / meds
metropolitan / amitryptiline
melds / meds
Give / Giv
Sari's / SSRI's
COD / OCD
pixel / paxil
pixel / paxil
Expire / Lexapro
SS RI / SSRI
effect / effexor
melds / meds
Streamline / Sertraline
Streamline / Sertraline
Streamline / Sertraline
Streamline / Sertraline
Streamline / Sertraline
Streamline / Sertraline
Streamline / Sertraline
ND / nd
TH / th
TH / th
Se rt / Sert
TH / th
Streamline / Sertraline
Streamline / Sertraline
Lu pin / Lupin
Streamline / Sertraline
TVA / Teva
TVA / Teva
TVA / Teva
TVA / Teva
Streamline / Sertraline
TVA / Teva

TVA / Teva
TVA / Teva
Streamline / Sertraline
Streamline / Sertraline
Streamline / Sertraline
Green stone / Greenstone
TVA / Teva
Streamline / Sertraline
Male / Mal
Styron / Setrona
Styron / Setrona
Styron / Setrona
ND / nd
/ fibromyalgia
Styron / Setrona
among st / amongst
Avian / Ativan
melds / meds
story's / storys
synth oms / synthoms
nickles / nuckles
celebs / celexa
cymbal ta / cymbalta
posses ed / possesed
melds / meds
ADD / adhd
biz / bcuz
sear / ser
melds / meds
Wendell / penndell
sq / siq
Monday / monday
melds / meds
PT SD / PTSD
diarrhea / diarrhoea
Saris / SSRIs
melds / meds

melds / meds
Carolann's / CarolAnn's
Carolann / CarolAnn
Effector / Effexor
effector / effexor
Homonym / Hmmmmm
Effector / Effexor
melds / meds
Amid / Amd
cine / c'cing
Effector / Effexor
verse / versa
Effector / Effexor
Effector / Effexor
Effector / Effexor
Effector / Effexor
Effector / Effexor
Effector / Effexor
effector / effexor
melds / meds
Effector / Effexor
effector / effexor
pixel / paxil
melds / meds
crab / carb
Effector / Effexor
effector / effexor
apex / arrapax
Effector / Effexor
HM / Hmm
ID / idk
dint / dont
excessive / excersice
Your / YOur
dint / dont
that / thtat
storyline / sertraline

trip tans / triptans
Storyline / Sertraline
sager / suger
melds / meds
cabs / carbs
Effector / Effexor
cabs / carbs
cardie / cardio
melds / meds
cabs / carbs
cabs / carbs
melds / meds
melds / meds
on line / online
melds / meds
vie / ve
Pixel / Paxil
melds / meds
Th's / th's
Expire / Lexapro
Expire / Lexapro
cabs / carbs
Saris / SSRIs
El / el
Dave / dave
melds / meds
Prue / pre
melds / meds
Pyrex / Zyprexia
crab / carb
woes / woss
terrify es / terrifyes
Min's / mins
EX / EFX
apt / appt
COD / OCD
Bu spar / Buspar

Indra / Inderal
excesses / excersise
Hm mm / Hmmm
Effector / Effexor
Effector / Effexor
Effector / Effexor
Effector / Effexor
cardie / cardio
Effector / Effexor
apatite / appatite
alto / alot
Miltie / milti
Effector / Effexor
protean / prothiaden
do ind / doind
Siaris / SSRIs
cabs / carbs
neuron tin / neurontin
NYC / nyc
dough nuts / doughnuts
PT SD / PTSD
cardie / cardio
Dr's / drs
weltering / wellbutrin
melds / meds
melds / meds
melds / meds
metropolitan / amitryptiline
from / frm
melds / meds
Siaris / SSRIs
COD / OCD
pixel / Paxil
pixel / Paxil
Expire / Lexapro
SS RI / SSRI
effector / effexor

melds / meds
you / yhou
storyline / sertraline
storyline / sertraline
storyline / sertraline
storyline / sertraline
storyline / sertraline
storyline / sertraline
storyline / sertraline
ND / nd
Th / th
Th / th
Se rt / Sert
Th / th
storyline / sertraline
storyline / sertraline
storyline / sertraline
TVA / Teva
TVA / Teva
TVA / Teva
TVA / Teva
storyline / sertraline
TVA / Teva
TVA / Teva
TVA / Teva
storyline / sertraline
storyline / sertraline
storyline / sertraline
Green stone / Greenstone
TVA / Teva
storyline / sertraline
Styron / Setrona
Styron / Setrona
Styron / Setrona
ND / nd
Styron / Setrona
BM / bpm

Avian / Ativan
melds / meds
anthems / synthoms
 / fibromyalgia
frost / first
Cele's / celexa
bu spur / buspur
cymbal ta / cymbalta
melds / meds
Ur / ur
ADD / adhd
COD / ocd
biz / bcuz
frost / first
see / ser
melds / meds
pernell / penndell
Sq / siq
iowas / iwas
melds / meds
PS TD / PSTD
 / diarrhea
Siaris / SSRIs
melds / meds
melds / meds

7 Aspell Spell Checker with medical dictionary

Note: the result is presented in the form "after-correction / before-correction", and if no spelling suggestion is provided from this system, the after-correction part will be left in blank.

7.1 Corrected appropriately (353 spelling errors)

guaranty / guarranty

Zoloft / zoloft
working / workng
Zoloft / zoloft
I'll / i'll
Zoloft / zoloft
Zoloft / zoloft
Definitely / Definetly
Zoloft / zoloft
didn't / didnt
Dr / dr
Dr / dr
couldn't / couldnt
Dr / dr
similar / similiiar
Dr / dr
Dr / dr
hadn't / hadnt
wouldn't / wouldnt
prescribed / perscribed
Dr / dr
approaching / aproaching
psychologist / spycologist
old's / olds
stiff / stuiff
separate / seperate
concerns / concernsm
wan / wasn
hygiene / hygeine
ridiculously / rediculously
Serotonin / Seratonin
Dopamine / dopamine
Zoloft / zoloft
apex / arrapax
Zoloft / zoloft
episodes / epsiodes
Your / YOur
that / thtat

Serotonin / Seratonin
psychiatrist / prychiatrist
give / giv
yourself / yoursel
Zoloft / zoloft
Zoloft / zolof
Zoloft / zolof
concern / conern
Zoloft / zoloft
acupuncture / accupunture
caused / cuased
Zoloft / zoloft
Zoloft / zoloft
research / reserach
Zoloft / zoloft
Zoloft / zoloft
Zoloft / zoloft
Zoloft / zoloft
beginning / beggining
Zoloft / zoloft
Zoloft / zoloft
exercising / excersizing
Zoloft / Zolof
Zoloft / Zolof
Zoloft / zoloft
Zoloft / zoloft
Zoloft / zoloft
weight loss / weightloss
Zoloft / zoloft
Zoloft / zoloft
habit / habbit
Zoloft / zoloft
Zoloft / zoloft
Zoloft / zoloft
Zoloft / zoloft
Zoloft / zoloft
Zoloft / zoloft

Zoloft / zoloft
Zoloft / zoloft
excesses1 / excersise
I've / i've
I've / Ive
beginning / beggining
Dr / dr
Zoloft / zoloft
Zoloft / zoloft
weight / weightt
Zoloft / zoloft
Zoloft / zoloft
Zoloft / zoloft
Zoloft / zoloft
I've / i've
Zoloft / zoloft
I'm / i'm
I'm / i'm
I'm / i'm
Zoloft / zoloft
I'll / i'll
Zoloft / zoloft
posted / psoted
Theanine / theanine
Theanine / theanine
Effexor / effexor
Effexor / effexor
Effexor / effexor
Effexor / effexor
Effexor / effexor
Sertraline / sertraline
Sertraline / sertraline
from / frm
Prozac / prozac
Prozac / prozac
I'm / i'm
daily / dailyh

Prozac / prozac
little / littlw
thou / yhou
Sertraline / sertraline
Jessica / jessica
Zoloft / zoloft
Zoloft / zoloft
Zoloft / zoloft
Zoloft / zoloft
Zoloft / zoloft
pharmaceutical / pharmacutical
Zoloft / zoloft
Dr / dr
generic / genereic
occasions / occassions
committing / comitting
Dr / dr
Zoloft / zoloft
initially / iniitally
obviously / obviosuly
Zoloft / zoloft
die in / diein
joint pain / jointpain
anuses / nauses
chores / choures
because / because
hyperthyroidism / hiperthyriodism
Theanine / theanine
because / becuase
because / becuase
doesn't / doesnt
neutrino / neoutrin
weird / wierd
Prozac / prozac
suicidal / sucidal
true / tru
Ur / ur

Zoloft / zoloft
because / becuase
COD / ocd
overwhelming / overwellming
didn't / didnt
suicidal / sucidal
Effexor / effexor
Effexor / effexor
Effexor / effexor
Effexor / effexor
didn't / didnt
spouse / sopouse
serveral / severl
couldn't / couldnt
exist / exsist
Zoloft / zoloft
Zoloft / zoloft
Theanine / theanine
Theanine / theanine
because / becuase
Zoloft / zoloft
Zoloft / zoloft
vengeance / vengance
Sertraline / sertraline
Zoloft / zoloft
don't / dont
hat's / thats
hat's / thats
hat's / thats
Neurontin / neurontin
apt / appt
in case / incase
any moment / anymoment
that's / thats
walk in / walkin
Zoloft / zoloft
Zoloft / zoloft

episodes / epsiodes
that's / thats
that's / thats
health care / healthcare
serotonin / seretonin
that's / thats
psychiatrist / prychiatrist
give / giv
Theanine / theanine
Theanine / theanine
Paxil / paxil
yourself / yoursel
Zoloft / zoloft
Zoloft / zoloft
Zoloft / Zoloft
concern / conern
Zoloft / zoloft
Neurontin / neurontin
acupuncture / accupunture
caused / cuased
Zoloft / zoloft
Zoloft / zoloft
Sertraline / sertraline
research / reserach
Zoloft / zoloft
Zoloft / zoloft
Zoloft / zoloft
Zoloft / zoloft
Effexor / effexor
Effexor / effexor
Effexor / effexor
Effexor / effexor
beginning / beggining
Zoloft / zoloft
Zoloft / zoloft
exercising / excersizing
Zoloft / Zolof

Zoloft / Zolof
Zoloft / zoloft
Zoloft / zoloft
Zoloft / zoloft
Theanine / theanine
weight loss / weightloss
Zoloft / zoloft
Zoloft / zoloft
habit / habbit
Zoloft / zoloft
Zoloft / zoloft
Dr / dr
Zoloft / zoloft
Zoloft / zoloft
Zoloft / zoloft
Zoloft / zoloft
Effexor / effexor
Zoloft / zoloft
Zoloft / zoloft
I've / i've
beginning / beggining
Zoloft / zoloft
Zoloft / zoloft
weight / weigtht
Zoloft / zoloft
Zoloft / zoloft
Paxil / paxil
Paxil / paxil
change / changed
Theanine / theanine
Zoloft / zoloft
Zoloft / zoloft
I've / i've
Zoloft / zoloft
I'm / i'm
I'm / i'm
I'm / i'm

Zoloft / zoloft
I'll / i'll
Zoloft / zoloft
posted / psoted
make / dmake
Give / Giv
Prozac / prozac
Prozac / prozac
Effexor / effexor
Effexor / effexor
I'm / i'm
Sertraline / sertraline
Sertraline / sertraline
daily / dailyh
Prozac / prozac
little / littlw
Theanine / theanine
Theanine / theanine
Jessica / jessica
Paxil / paxil
Zoloft / zoloft
Zoloft / zoloft
Zoloft / zoloft
Zoloft / zoloft
Zoloft / zoloft
pharmaceutical / pharmacutical
Zoloft / zoloft
Lu pin / Lupin
Dr / dr
don't / dont
generic / genereic
occasions / occassions
committing / comitting
Dr / dr
Zoloft / zoloft
initially / iniitally
obviously / obviosuly

Effexor / effexor
Effexor / effexor
Effexor / effexor
in case / incase
different / diffrent
storys / stories
Zoloft / zoloft
dieing / diein
joint pain / jointpain
anuses / nauses
Theanine / theanine
chores / choures
I've / ive
Sertraline / sertraline
because / becuase
don't / dont
Paxil / paxil
Paxil / paxil
hyperthyroidism / hiperthyroidism
any moment / anymoment
knuckles / nuckles
because / becuase
Effexor / effexor
Effexor / effexor
because / becuase
I've / ive
Prozac / prozac
neutrino / neoutrin
weird / wierd
different / diffrent
don't / dont
suicidal / sucidal
possessed / tposseded
tru / true
Zoloft / zoloft
because / becuase
overwhelming / overwellming

didn't / didnt
didn't / didnt
suicidal / sucidal
walk in / walkin
Monday / monday
suppose / sopouse
several / serverl
couldn't / counldnt
hands / hads
because / becuase
exist / exsist
Zoloft / zoloft
Zoloft / zoloft
Zoloft / zoloft
vengeance / vengance
Zoloft / zoloft
yourself / urself
yourself / urself
don't / dont

7.2 Corrected inappropriately (211 spelling errors)

dint / dont
thee / ther
to days / todays
dint / dont
excessive / excersice
apatite / appatite
dint / dont
diff rent / diffrent
Ive / ive
dint / dont
frost / frist
Ive / ive
diff rent / diffrent
dint / dont
frost / frist
Iowas / i was
hades / hads

itself / urself
itself / urself
dint / dont
melds / meds
melds / meds
melds / meds
ha ha / haha
melds / meds
melds / meds
US / ujs
melds / meds
melds / meds
melds / meds
melds / meds
melds / meds
melds / meds
melds / meds
over analyzing / overanalyzing
ha ha / haha
melds / meds
Nurse girl / Nursegirl
2ND / 2nd
melds / meds
catastrophes / catastrophize
MG / OMG
drys / drs
alto / alot
melds / meds
CZ / cuz
melds / meds
melds / meds
drys / drs
melds / meds
Mg's / mgs
Melds / Meds
CYST / CYS
judge mental / judgememtal

em erg / emerg
SO / sooooo
of fence / offence
melds / meds
Lent / learnt
crab / carb
mamma's / mammo's
melds / meds
int ital / intital
Lola / lol
ZR / XR
Lola / lol
Homonym / Hmmmmm
Carol Ann's / CarolAnn's
Carol Ann / CarolAnn
Homonym / Hmmmmm
melds / meds
AMD / Amd
cine / c'ing
verse / versa
melds / meds
melds / meds
crab / carb
ID / idk
sager / suger
melds / meds
melds / meds
melds / meds
melds / meds
melds / meds
vie / ve
melds / meds
Th's / th's
Eli / el
Dave / dave
Dry's / Dr's
melds / meds

pare / pre
melds / meds
ca lender / calender
crab / carb
terrify es / terrifyes
Min's / mins
EX / EFX
Hmm / Hmmm
alto / alot
mil ti / milti
do ind / doind
NYC / nyc
drys / drs
melds / meds
melds / meds
damage / dmake
melds / meds
melds / meds
Give / Giv
melds / meds
ND / nd
TH / th
TH / th
Se rt / Sert
TH / th
Green stone / Greenstone
Male / Mal
ND / nd
among st / amongst
melds / meds
story's / storys
synth oms / synthoms
nickles / nuckles
posses ed / possesed
melds / meds
biz / bcuz
sear / ser

melds / meds
sq / siq
Monday / monday
melds / meds
melds / meds
melds / meds
Carolann's / CarolAnn's
Carolann / CarolAnn
Homonym / Hmmmmm
melds / meds
cine / c'cing
verse / versa
melds / meds
melds / meds
crab / carb
apex / arrapax
HM / Hmm
ID / idk
dint / dont
excessive / excersice
Your / YOur
dint / dont
that / thtat
sager / suger
melds / meds
cardie / cardio
melds / meds
melds / meds
melds / meds
on line / online
melds / meds
vie / ve
melds / meds
Th's / th's
El / el
Dave / dave
melds / meds

Prue / pre
melds / meds
woes / woss
terrify es / terrifyes
Min's / mins
EX / EFX
apt / appt
excesses / excersise
Hm mm / Hmmm
apatite / appatite
alto / alot
Miltie / milti
do ind / doind
cabs / carbs
NYC / nyc
dough nuts / doughnuts
Dr's / drs
melds / meds
melds / meds
melds / meds
from / frm
melds / meds
melds / meds
you / yhou
ND / nd
Th / th
Th / th
Se rt / Sert
Th / th
Green stone / Greenstone
ND / nd
melds / meds
anthems / synthoms
frost / first
bu spur / buspur
melds / meds
Ur / ur

biz / bcuz
frost / first
see / ser
melds / meds
Sq / siq
iowas / iwas
melds / meds
melds / meds
melds / meds

8 Jazzy Spell Checker with medical dictionary

Note: the result is presented in the form “after-correction / before-correction”, and if no spelling suggestion is provided from this system, the after-correction part will be left in blank.

8.1 Corrected appropriately (240 spelling errors)

guaranty / guarranty
Wart / Wort
I / i
us / ujs
working / workng
I / i
I / i
I'll / i'll
I / i
I / i
Initial / Initially
startup / start-up
Definitely / Definitly
didn't / didnt
couldn't / couldnt
similar / similiar
hadn't / hadnt
wouldn't / wouldnt

consoler / counselor
don't / dont
psyche / psych
Zoloft / zoloft
Zoloft / zoloft
prescribed / perscribed
old / olds
stuff / stuiff
the / ther
separate / seperate
concerns / concernsm
fever / favor
emerge / emerg
so / sooooo
can't / cant
hygiene / hygeine
sugar / sugary
correct words:
I / i
episodes / epsiodes
don't / dont
exercise / excersice
that / thtat
psychiatrist / prychiatrist
give / giv
yourself / yoursel
concern / conern
caused / cuased
research / reserach
calendar / calender
terrifies / terrifyes
exercise / excersise
I've / i've
I've / Ive
weight / weigtht
doing / doind
I / i

Zoloft / zoloft
Zoloft / zoloft
I / i
I / i
I / i
I / i
I / i
I / i
I / i
wean / ween
I've / i've
I'm / i'm
I'm / i'm
I'm / i'm
I / i
I'm / i'm
I / i
I / i
Zoloft / zoloft
Zoloft / zoloft
Zoloft / zoloft
I'll / i'll
posted / psoted
I / i
make / dmake
Give / Give
popped / poopped
popped / poopped
pop / poop
I'm / i'm
I / i
I / i
daily / dailyh
I / i
I / i
I / i
little / littlw

you / yhou
neurologists / neurologist
weaning / weening
I / i
don't / dont
occasions / occassions
committing / comitting
I / i
I / i
I / i
I / i
Zoloft / zoloft
Zoloft / zoloft
Zoloft / zoloft
Zoloft / zoloft
psyche / psych
I / i
obviously / obviosuly
I / i
different / diffrent
I / i
I / i
I / i
Zoloft / zoloft
Zoloft / zoloft
I / i
I / i
I / i
I / i
I / i
I / i
I / i
Zoloft / zoloft
I / i
I / i
I / i
I / i

Effexor / effexor
because / becuase
I / i
don't / dont
I / i
Effexor / effexor
I / i
I / i
I / i
Zoloft / zoloft
Zoloft / zoloft
I / i
I / i
I / i
I / i
first / frist
I / i
because / becuase
I / i
because / becuase
doesn't / doesnt
neutrino / neutrin
weird / wierd
different / diffrent
I / i
I / i
don't / dont
Zoloft / zoloft
Zoloft / zoloft
suicidal / sucidal
Effexor / effexor
I / i
I / i
I / i
I / i
I / i
I / i

I / i
possessed / possesed
I / i
I / i
I / i
what's / whats
Zoloft / zoloft
Zoloft / zoloft
Zoloft / zoloft
Effexor / effexor
Effexor / effexor
because / becuase
overwhelming / overwellming
I / i
I / i
I / i
I / i
I / i
first / frist
I / i
didn't / didnt
I / i
didn't / didnt
suicidal / sucidal
walking / walkin
Monday / monday
several / severl
I / i
I / i
I / i
because / becuase
I / i
true / tur
mediation / medication
I / i
I / i
I / i

Effexor / effexor
Effexor / effexor
what's / whats
because / becuase
overwhelming / overwellming
I / i
I / i
I / i
I / i
I / i
first / frist
I / i
Effexor / effexor
Effexor / effexor
didn't / didnt
I / i
didn't / didnt
suicidal / sucidal
walking / walkin
Monday / monday
was / iwas
several / severl
I / i
I / i
I / i
Effexor / effexor
Effexor / effexor
Effexor / effexor
because / becuase
I / i
I / i
exist / exsist
nose / nausea
vengeance / vengance
I / i
I / i
don't / dont

8.2 Corrected appropriately (334 spelling errors)

Ha / Hi

me / .m

meads / meds

meads / meds

/ chill-like

alight / alright

generalist / generalized

Sat / St

pa / pm

meads / meds

Dry / Dr

Dry / Dr

Dry / Dr

mug /mg

/ haha

hone / hon

meads / meds

meads / meds

Recast / Rx'd

mug / mg

Xx / Rx

dock/ doc

dock/ doc

meads / meds

meads / meds

meads / meds

meads / meds

meads / meds

meads / meds

gone / gonna

/ judgment

hone / hon

meads / meds

/ overanalyzing

husband / housebound

/ haha

meads / meds
/ Nursegirl
redecorated / redecorate
/ everything's
redecorated / redecorate
meads / meds
catastrophes / catastrophize
dock / doc
unhelpfully / unhelpful
hrs / drs
dry / dr
dry / dr
dry / dr
alto / alot
meads / meds
cue / cuz
meads / meds
dry / dr
meads / meds
/ minimize
the / tho
dry / dr
hrs / drs
meads / meds
dry / dr
/ specializes
the / tho
approaching / aproaching
meads / meds
ax / ex
into / info
dock / doc
/ specializing
overcoat / overreact
ax / ex
ax / ex
ax / ex

ax / ex
ax / ex
into / info
into / info
/ non-judgemental
/ minimize
ax / ex
ax / ex
behaver / behavior
hrs / yrs
ax / ex
ax / ex
deadlier / toddler
/ hospitalized
/ practicing
ax / ex
/ ex-husband
alight / alright
ovens / offence
/ Discontinuation
meads / meds
learn / learnt
oiliest / whilist
Ha / Hi
crab / carb
crabs / carbs
sacks / snacks
mamma's / mammo's
meads / meds
entitle / intital
initial / initially
Adhesion / Watson
/ ridiculously
low / lot
low / lot
Dry / Dr
/ Hmmmmm

Carolina's / CarolAnn's
Carolina / CarolAnn
hone / hon
dry / Dr
/ Hmmmmm
meads / meds
do / d
A / c
amid / Amd
cuing / c'ing
oiliest / whilst
popped / pooped
crab / carb
airbags / arrapax
ham / Hmm
id / idk
/ healthcare
A / u
into / info
mug / mg
crabs / carbs
annal / Anna
ax / ex
figs / veggies
labs / lbs
intakes / intake
moss / mos
we / w
oh / o
vie / ve
be / b
beaconing / beggining
exercising / excersizing
boy's / body's
dive / dave
or's / Dr's
silver / sliver

per / pre
whittles / weightloss
attacked / it.Good
hrs / yrs
OS / oz
or / org
willower / willpower
habit / habbit
woos / woss
woe / Wow
/ marathon
hrs / hr
mains / mins
apt / appt
dry / dr
skin / skinny
mom / Hmmm
initial / initially
alto / alot
malt / milti
vis / vs
years / years.I
stablest / stablized
labs / lbs
flake / fluke
labs / lbs
labs / lbs
whack / wack
crabs / carbs
hrs / yrs
/ nyc-please
be / b
A / c
/ empathize
Jean / Jan
/ dosage
mug / mg

figs / veggies
sympathies / sympathize
/ mediation
/ insatiable
Iran / Iraq
/ front-line
labs / lbs
Initial / Initially
labs / lbs
labs / lbs
woe / wow
we / w
oh / o
hrs / drs
we / w
oh / o
mug / mg
mug / mg
erects / workouts
Hi / Ha
Kindle / Kendall
Ha / Hi
meads / meds
hrs / yrs
meads / meds
meads / meds
A / u
re / r
/ materialistic
A / u
re / r
A / u
re / r
A / u
A / u
sos / sis
A / u

re / r
A / u
A / u
farm / frm
A / u
A / u
A / u
be / b
meads / meds
type / typo
/ dosage
ha / hi
be / bk
be / bk
meads / meds
/ jessica
xi / x
Ha / Hi
/ zoloft-induced
hrs / yrs
vis / vs
time / time-I
yoke / yoga
Sort / Sert
/ hurtful
behaver / behavior
tear / teary
/ irritable
colon / color
dry / dr
generic / generics
Hi / Hi
/ runaround
/ FDA-speak
tap / http
me / m
come / com

buttock / biotech
tap / http
/ www
/ peoplespharmacy
come/ com
pep / php
cupping / upping
/ dosage
/ brand-name
/ Greenstone
Mail / Mal
generic / generics
/ Approx
dry / dr
innately / iniitally
bum / bpm
worse / worsen
/ mediation
boy's / body's
inks / incase
meads / meds
woe / wow
story's / storys
deign / diein
/ jointpain
chores / choures
iv / ive
/ synthoms
opposite / website
/ anymoment
she / sh**
knuckles / nuckles
iv / ive
/ buspur
yoke / yoga
opposite / website
meads / meds

/ discontinuation
true / tru
dock / doc
ha / hi
our / ur
add / adhd
cod / ocd
bucks / bcuz
sear / ser
meads / meds
sit / siq
intakes /intake
was / iwas
spouse / sopouse
/ counldnt
heads / hads
/ discontinuation
dock / doc
ha / hi
our / ur
add / adhd
cod / ocd
bucks / bcuz
sear / ser
meads / meds
sit / siq
intakes / intake
spouse / sopouse
/ counldnt
heads / hads
opposites / websites
meads / meds
meads / meds
ourself / urself
A / u
ourself / urself
/ actionable

I / i
we / e
